# Common Fragile Sites Are Characterized by Faulty Condensin Loading after Replication Stress

**DOI:** 10.1016/j.celrep.2020.108177

**Published:** 2020-09-22

**Authors:** Lora Boteva, Ryu-Suke Nozawa, Catherine Naughton, Kumiko Samejima, William C. Earnshaw, Nick Gilbert

**Affiliations:** 1 MRC Human Genetics Unit, The University of Edinburgh, Crewe Rd South, Edinburgh EH4 2XU, UK; 2 Wellcome Centre for Cell Biology, The University of Edinburgh, Michael Swann Building, Max Born Crescent, Edinburgh EH9 3BF, UK

**Keywords:** chromosome, chromatin, condensin, replication, replication stress, genome stability, condensation, common fragile sites

## Abstract

Cells coordinate interphase-to-mitosis transition, but recurrent cytogenetic lesions appear at common fragile sites (CFSs), termed CFS expression, in a tissue-specific manner after replication stress, marking regions of instability in cancer. Despite such a distinct defect, no model fully provides a molecular explanation for CFSs. We show that CFSs are characterized by impaired chromatin folding, manifesting as disrupted mitotic structures visible with molecular fluorescence *in situ* hybridization (FISH) probes in the presence and absence of replication stress. Chromosome condensation assays reveal that compaction-resistant chromatin lesions persist at CFSs throughout the cell cycle and mitosis. Cytogenetic and molecular lesions are marked by faulty condensin loading at CFSs, a defect in condensin-I-mediated compaction, and are coincident with mitotic DNA synthesis (MIDAS). This model suggests that, in conditions of exogenous replication stress, aberrant condensin loading leads to molecular defects and CFS expression, concomitantly providing an environment for MIDAS, which, if not resolved, results in chromosome instability.

## Highlights

• Cytogenetic lesions appear at common fragile sites (CFSs) after replication stress
• CFSs have impaired chromatin folding visible with molecular FISH probes
• CFS lesions are marked by faulty condensin loading
• Condensin depletion increases the frequency of CFS lesions

## Graphical Abstract

**Figure:**
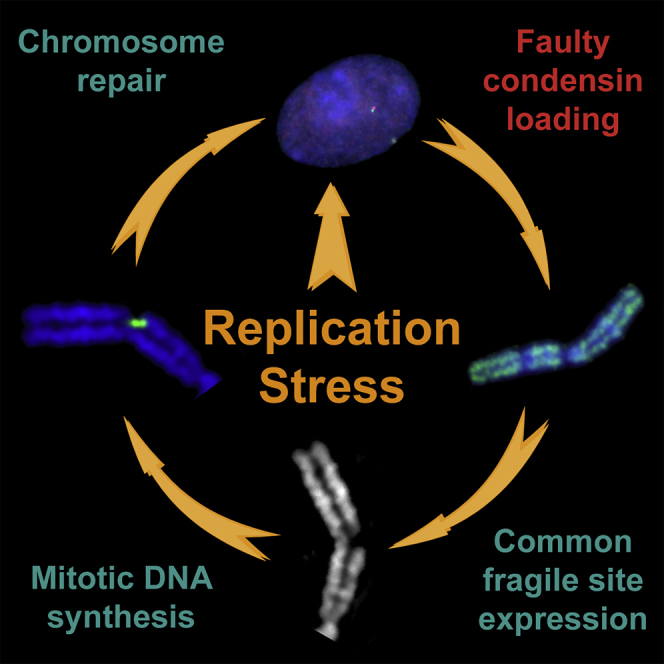

## Teaser

Common fragile sites are genomic regions that exhibit chromatin-folding defects in conditions of exogenous replication stress. Using FISH, Boteva et al. show those sites also have altered chromatin architecture in unperturbed conditions and show faulty condensin loading, which leads to chromatin-folding defects and mitotic DNA synthesis.

## Introduction

The folding of chromosomes in preparation for mitosis is the most profound structural change the genome undergoes throughout a cell’s lifetime (Antonin and Neumann, 2016). Mitotic condensation is linked to successful cell division and cell cycle progression in a functional and regulatory manner, and its failure can be costly, leading to lagging chromosomes and aneuploidy (Gordon et al., 2012; Saldivar et al., 2018; Zhu et al., 2018). Much effort has been made to define the molecular basis of the condensation process and to bridge the cytogenetic features of mitotic chromosomes with molecular-level understanding of the chromatin and scaffolding proteins that comprise them. As a result, it is now accepted that a fully folded, cytogenetically normal metaphase chromosome is the product of successful and timely completion of inter-connected processes, including replication, sister chromatid separation, and chromatin condensation (Gibcus et al., 2018; Ono et al., 2013; Wechsler et al., 2011). Consequently, the cytogenetic integrity of chromosomes is affected by the disruption of these processes; common fragile sites (CFSs) (Durkin and Glover, 2007), regions of the genome known for forming lesions on metaphase chromosomes when cells are challenged with replication stress (Zeman and Cimprich, 2014), in a process called CFS expression, are a prominent example. Illustrating the importance of mitotic compaction for genome stability, these sites overlap with recurrent cancer deletions and tumor suppressor genes frequently lost in cancer (Bignell et al., 2010; Negrini et al., 2010; Le Tallec et al., 2013). Unlike constitutively fragile locations, such as fragile X, CFSs form in a cell-type-specific manner, leading to suggestions that an epigenetic component has a role in their fragility (Letessier et al., 2011; Le Tallec et al., 2011). A number of factors have been identified, including late replication timing, transcription of long genes, and features of the underlying DNA sequence (Blin et al., 2019; Brison et al., 2019; Fungtammasan et al., 2012; Helmrich et al., 2011; Maccaroni et al., 2020; Okamoto et al., 2018; Le Tallec et al., 2014; Wei et al., 2016; Wilson et al., 2015). CFSs also require FANCD2 for efficient replication (Madireddy et al., 2016; Pentzold et al., 2018) and have been identified as regions in which active DNA synthesis is apparent on mitotic chromosomes in a process dependent on POLD3 and the Mus81 nuclease (Minocherhomji et al., 2015). The steps involved in triggering synthesis remain unknown, but they also require the TRAIP ubiquitin ligase (Sonneville et al., 2019). Recent high-resolution mapping of mitotic DNA synthesis (MIDAS) (Macheret et al., 2020) has shown that mitotic synthesis occurs at genomic locations measuring up to 1.2 Mb in size, overlapping with previously identified CFSs. Assessment of replication dynamics at those sites confirms that CFSs take a long time to replicate and remain unreplicated in late S phase under conditions of replication stress.

Although the replication states of the CFS regions have recently been characterized, there is little mechanistic insight into how replication defects lead to the condensation defects observed on mitotic chromosomes. Condensation defects have also been shown to underlie homologous recombination (HR)-deficiency-mediated mitotic lesions and, if not resolved, lead to DNA damage and chromosomal instability (Chan et al., 2018). The effectors of such condensation failures are likely to be proteins that drive mitotic folding, such as the condensin I and II complexes, which are crucial for chromosome compaction (Gibcus et al., 2018; Lipp et al., 2007; Samejima et al., 2012). Furthermore, mechanisms established in yeast show that the post-replicative chromatin state is monitored by the ATR homolog Mec1; in the absence of Mec1, a subset of genomic locations, including slow replicating zones, which resemble CFSs, become sensitive to mitotic condensation and develop breaks in a condensin-dependent manner (Cha and Kleckner, 2002; Hashash et al., 2012).

Given the close relationship between replication and mitotic compaction (Ono et al., 2013), we hypothesized that disrupted mitotic folding may arise as a consequence of replication stress at sensitive regions, such as CFSs. Using a fluorescence *in situ* hybridization (FISH)-based approach, we show that CFSs are characterized by failure of local chromatin to compact for mitosis; this is not only the case at cytogenetic lesions but also at sites that appear cytogenetically normal, and we demonstrate a previously unknown propensity for smaller-scale molecular lesions (100 kb), visible only at the molecular (imaged by FISH), and not the cytogenetic, level. We show that molecular and cytogenetic instability at CFSs is dependent on condensin and remodels chromatin at the G2/M boundary to facilitate mitotic folding. Analysis of condensin complexes indicates that condensin I, rather than condensin II, is the effector of disrupted mitotic compaction at CFSs. Our model suggests that, after replication, non-fragile regions undergo structural and compositional “priming” of chromatin in preparation for mitosis. In contrast, CFSs are regions of the genome in which, even in unperturbed conditions, chromatin is inefficiently “primed” for mitotic compaction, likely because of delayed replication or the presence of post-replicative intermediates, which can be resolved by extending the duration of G2. CFSs are characterized by aberrant condensin loading, leading to molecular lesions, and in the extreme conditions of exogenous replication stress, cytological chromosome abnormalities are apparent.

## Results

### CFS Frequency and Repertoire in RPE1 and HCT116 Cells

**Figure 1:**
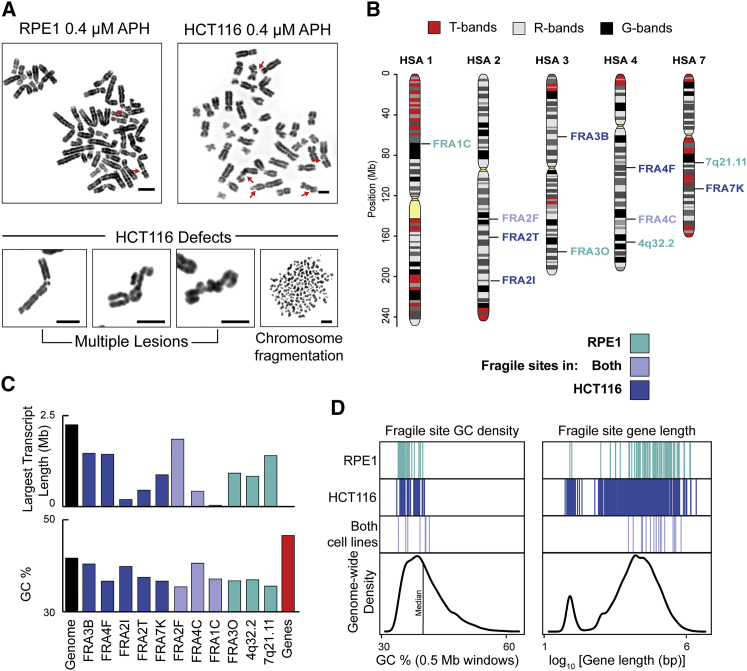
Characterization of CFSs in HCT116 and RPE1 Epithelial Cells (A) Representative metaphase spreads (reverse DAPI banding) from RPE1 (left) and HCT116 (right) cell lines, showing CFS fragility (red arrows) after aphidicolin (APH) treatment (top); bottom, extreme chromosomal defects in HCT116 cells; Scale bar, 5 μm. (B) Ideograms showing most frequent APH-dependent common fragile site locations in RPE1 and HCT116 epithelial cells, cytogenetically scored by DAPI banding. CFSs specific to HCT116 cells (blue), RPE1 (green), and both (mauve) are indicated. (C) Length of largest transcript (top) and GC content (bottom) at sites fragile in HCT116 (blue), RPE1 (green), or both cell lines (mauve). (D) Left, genome-wide GC (percentage in 0.5-Mb windows) density plot with GC density at CFSs in HCT116 (blue), RPE1 (green) or both cell lines (mauve). Right, genome-wide gene-length (NCBI genes) density plot with gene length of genes encompassed within CFSs in HCT116 (blue), RPE1 (green), or both cell lines (mauve). See also Figure S1 and Table S1.

To analyze the relationship between chromosome architecture and CFS structure, we characterized the CFS repertoire and frequency in two epithelial chromosomally near-normal diploid cell lines (HCT116 and RPE1), using DAPI banding, after inducing replication stress with aphidicolin (APH); 372 lesions across 371 metaphases for APH concentrations ranging from 0.1 to 0.6 μM were observed, showing that greater APH concentration led to increased breakage rates and more-severe CFS phenotypes (Figures S1A and S1B), with a concomitantly delayed cell cycle (Figure S1C). Cytogenetic lesions were mapped and scored in metaphase spreads prepared from HCT116 (n = 94) and RPE1 (n = 64) cells after 24-h of treatment with 0.4 μM APH (Figures 1A, 1B, S1D, and S1E; Table S1). Despite both cell lines being of epithelial origin, the CFS repertoire differed significantly: FRA3B was the most fragile site in the HCT116 line (23% of all breaks), followed by locations on chromosome 2 (FRA2I, 2q33.2; FRA2T, 2q24.1). In contrast, the most fragile location in the RPE1 cell line, FRA1C on 1p31.2, was only weakly fragile in HCT116 (18.6% of all breaks in RPE1; 5.8% in HCT116); additionally, 4q32.2, one of the most common break sites (∼10% of all breaks) in the RPE1 cell type, has not been previously identified as a CFS location, although it was observed once in a previous study (Mrasek et al., 2010). A previous analysis of CFS distribution in HCT116 cells (Le Tallec et al., 2013) also indicated that FRA3B was the most common site, but there were also differences: in our study, FRA4F and FRA2I instability was more frequent, whereas FRA4D and FRA16D instability was not readily apparent. In contrast, a further study found that FRA16D was the most common fragile site in HCT116 cells (Hosseini et al., 2013), indicating differences in CFS repertoire and frequency among sub-clones.

CFSs are reported to share a number of structural characteristics: the presence of long genes, AT-rich sequences and late replication timing (Arlt et al., 2009; Fungtammasan et al., 2012; Wilson et al., 2015). The genomic features of the sites we identified were consistent with these trends, although CFSs do not contain the most guanine cytosine (GC)-poor regions in the genome, the longest genes (Figures 1C and 1D), or the latest replicating regions (Zhao et al., 2020) (Table S1). Among the most-fragile locations in our study, 9 of 11 overlapped with genes larger than 0.3 Mb, including FRA3B (FHIT), FRA4F (GRID2, CCSER), 4q32.2 (MARCH1, 0.85 Mb; FSTL5, 0.78 Mb), and FRA7E, which span MAGI2 (1.4 Mb). Although the frequent FRA1C site in the RPE1 cell line does not overlap with any long genes, it is near to LRRC7 (0.32 Mb) and 2.5 Mb away from NEGR1 (0.89 Mb). The Catalogue of Somatic Mutations in Cancer (COSMIC) mutation data also showed that, as expected, the majority of the most-frequent CFSs (8 of 11) overlapped with recurrent cancer-deletion clusters (Le Tallec et al., 2013).

### CFS Regions Have Irregular Chromatin Structures in the Absence of Replication Stress

**Figure 2:**
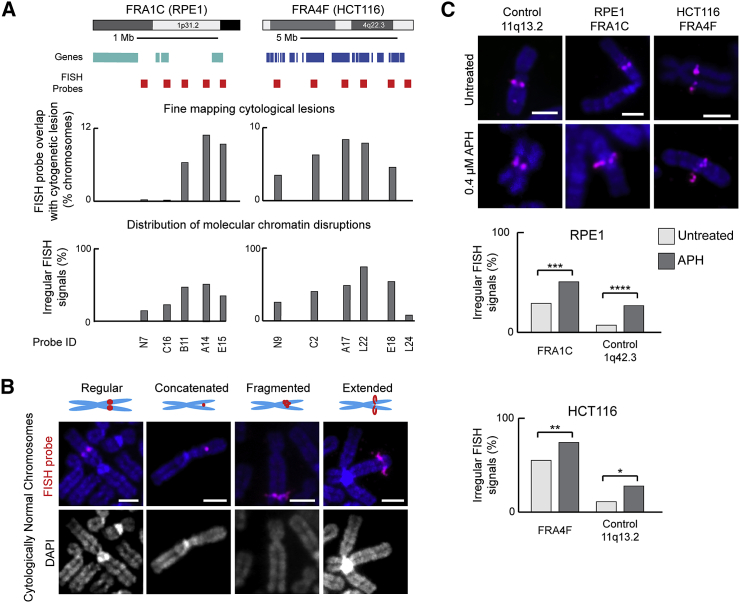
Irregular Chromatin Structures at CFSs in the Presence and Absence of APH-Induced Replication Stress (A) Quantification of FISH probe signals across FRA4F (HCT116 cells, n = 439) and FRA1C (RPE1 cells, n = 180) to finely map cytological lesions (top) and distribution of molecular chromatin disruptions (bottom). FISH probe IDs are described in the Method Details. (B) Irregular FISH probe phenotypes (magenta) on cytogenetically normal chromosomes. Regular, symmetrical signals; concatenated, a single-signal sitting between the two sister chromatids; fragmented, multiple, asymmetric signals; extended, a signal extending beyond the DAPI-stained chromosome area. Scale bar, 2.5 μm. (C) Chromosomes from untreated cells (top) or cells treated with APH (bottom), to induce replication stress, hybridized to FISH probes for a non-fragile locus 11q13.2 (probe ID: P21, n = 84 for HCT116, n = 87 for RPE1) or fragile-loci FRA4F (probe ID: A17; HCT116 cells, n = 123) and FRA1C (probe ID: A14; RPE1 cells, n = 146). Scale bar, 2.5 μm. Bottom, quantification of irregular FISH signals in the presence and absence of APH. p values for a χ^2^ test. ^∗^p < 0.05, ^∗∗^p < 0.001, ^∗∗∗^p < 0.001, ^∗∗∗∗^p < 0.0001. See also Figure S2 and Table S2.

Cytogenetic mapping after replication stress revealed a range of phenotypes at mitosis, including chromatid breaks and gaps, chromosome gaps, concatenations, and other complex abnormalities, with no relationship among particular locations and the abnormality observed (Figure S1E). Because cytogenetic mapping provides relatively low-resolution information on the molecular location of a fragile-site lesion a bacterial artificial chromosome (BAC)-walking strategy was used to fine-map five cytogenetically identified CFS regions (Table S1). Probes were selected spanning the sites, and the frequency of chromosomes showing cytogenetic lesions overlapping with the probes were quantified (Figures 2A and S2A). Rather than always occurring at the same location, breaks appeared across large genomic regions encompassing CFS sites: a high frequency of breaks were observed at a fragile “core” region, which tailed off at BACs located upstream or downstream (80% break overlap at the core of the sites reduced to 33% at the flanks). Fluorescent BAC signals were often observed to span CFS lesions, with the fluorescence intensity of the probes peaking over the DAPI faint regions, consistent with DNA being present within the cytogenetically visible breaks (Figure S2B).

To better characterize mitotic chromosome fragility, the chromatin state of CFSs was assessed with the FISH signal from the BAC probes as a marker for chromatin condensation, at a molecular level. After APH, treatment probes within CFS regions showed a propensity to have atypical FISH signals, even in the absence of cytogenetic lesions at the corresponding CFS site (Figures 2B and S2D). Rather than twin symmetric foci on mitotic chromosomes, CFS-spanning probes frequently formed multiple, asymmetric spots or appeared as a single spot sitting between the two chromatids. However, the most extreme of these atypical signals was a phenotype in which BACs extended away from the chromosome, spreading far beyond the DAPI-dense area. Although aberrant chromatin folding is often seen in FISH of mitotic chromosomes, under the conditions used for these studies, control loci rarely showed chromatin-compaction defects (Figure S2D). Instead, these signals are reminiscent of abnormal FISH signals formed at telomeres in response to replication stress, termed “fragile telomeres” (Sfeir et al., 2009), and are indicative of problems with mitotic condensation and decatenation.

To investigate how irregular FISH signals related to cytological fragility, the frequency of chromosomes showing such signals for each of the BAC probes was quantified (Figures 2A and S2A). This fine-mapping of molecular-level misfolding phenotype (i.e., irregular FISH signals) revealed that the frequency of these signals had a similar distribution along the CFS regions as that of the cytological breaks. A more extensive analysis at FRA1C and FRA4F indicated that the frequency of misfolding extended beyond the region most affected by cytogenetic lesions, and abnormal compaction signals were observed at BACs that did not frequently overlap with breaks (probes L24, N7, and C16 in Figure 2A). Furthermore, irregular FISH signals were observed at a similar frequency, irrespective of whether cytological lesions were present or absent (Figure S2C). This analysis revealed that CFS regions, although highly prone to forming cytogenetic abnormalities, are also characterized by an additional level of instability at a molecular level, indicative of a defect in mitotic chromosome condensation.

Because molecular misfolding was observed in chromosomes exposed to replication stress, we determined whether such signals were present in unperturbed cells, which do not show cytogenetic lesions. Signal phenotypes for two BACs at FRA1C and at FRA4F were examined, and surprisingly, molecular misfolding was elevated at fragile sites compared with control loci, even in the absence of replication stress (Figure 2C). This was particularly pronounced in HCT116 cells, in which 60% of chromosomes carried disruptions in FRA4F in the absence of replication stress and argues against these folding defects being caused by rare replication events. Conversely, to determine how replication stress affected mitotic condensation at non-CFS regions, the signal phenotypes for two control regions, located on human chromosome (HSA) 1q42.3 and HSA 11q13.2, were examined after APH treatment. The frequency of atypical signals increased at these non-fragile loci in the presence of replication stress but remained much lower compared with the CFS regions (7 to 27% in RPE1, 11% to 28% in HCT116) but demonstrate that replication stress, per se, can lead to an increase in the frequency of mitotic-condensation defects at typical genomic locations.

### Extending G2 Reduces Cytogenetic Lesions and Molecular Defects at CFS

**Figure 3:**
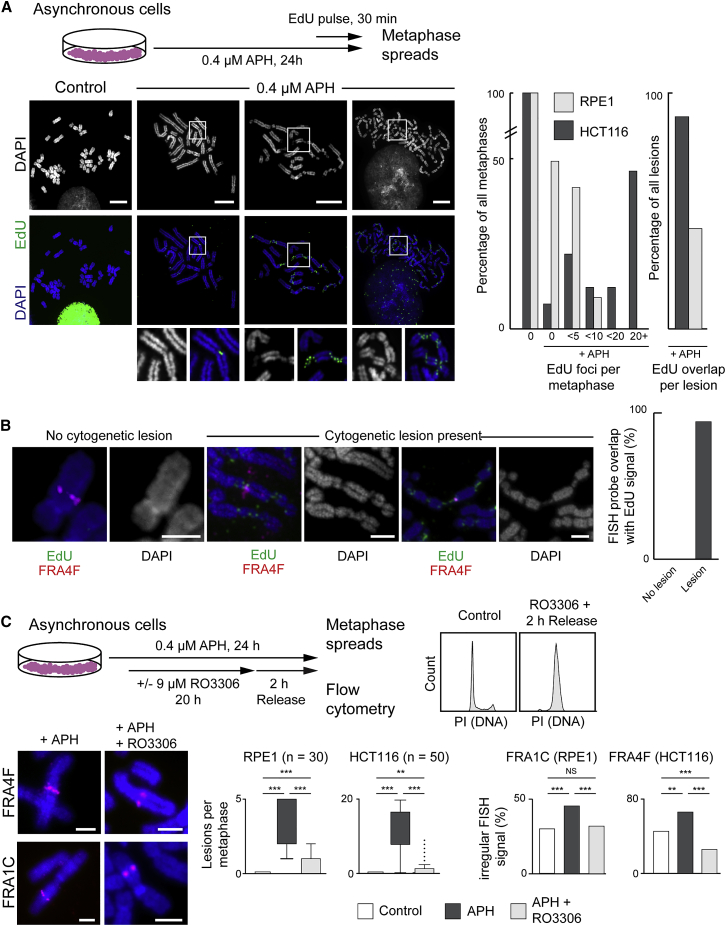
Mitotic DNA Synthesis (MIDAS) Is Coincident with Large-Scale Chromatin Disruptions at Cytologically Visible CFSs (A) Top, staining procedure for MIDAS visualized with EdU and FITC-azide. Bottom, left, representative metaphase spreads prepared from cells treated with or without APH. Insets show MIDAS (green signal) and widespread chromosome compaction defects. Right, quantification of MIDAS in RPE1 (n = 65) and HCT116 (n = 82) metaphase spreads from APH-treated cells and overlap between cytogenetic lesions and MIDAS in HCT116 (n = 1,622 foci) and RPE1 (n = 96 foci) cells. Scale bar, 10 μm. (B) Atypical FISH signals, cytogenetic lesions, and MIDAS after APH treatment at the FRA4F locus (probe ID: A17) in HCT116 cells. Representative chromosomes are shown, with increasing degrees of lesions and aberrant condensation. Right, graph showing overlap frequency of FRA4F probe with MIDAS foci on chromosomes in the presence or absence of cytogenetic lesions. Scale bars, 2.5 μm. (C) Top, left, treatment conditions for delaying G2 with RO3306 after induction of replication stress. Top, right, flow cytometry analysis of cells stained with propidium iodide to assess cell cycle stage. Bottom, left, representative images of FISH signals at the FRA4F (probe ID: A17; HCT116 cells) or FRA1C (probe ID: A14; RPE1) loci after APH treatment followed by a normal duration (left) or extended (right) G2. Bottom, middle, lesions per metaphase (boxplots; p values are for a Wilcoxon test); bottom, right, irregular FISH signals (bar graph; n = 84, 34, and 48 chromosomes per condition for RPE1 cells and n = 58, 49, and 70 chromosomes per condition for HCT116 cells; p values for a χ^2^ test) after APH treatment followed by a normal (dark gray) or delayed (light gray) G2. Scale bars, 2.5 μm. p values: NS, not significant; ^∗^p < 0.05, ^∗∗^p < 0.001, ^∗∗∗^p < 0.001, ^∗∗∗∗^p < 0.0001. See also Figure S3 and Table S2.

CFS regions are sites of DNA synthesis on metaphase chromosomes: by using a short pulse with the thymidine analog 5-ethynyl-2′-deoxyuridine (EdU) in mitosis, MIDAS foci can be observed at cytogenetic CFS lesions (Minocherhomji et al., 2015). To characterize the relationships among MIDAS, cytological lesion formation, and molecular misfolding in these cell lines, a similar labeling approach was used (Figure 3A). MIDAS occurred at DAPI-faint regions and, on many occasions, could be seen bridging gaps in chromosomes (Figures 3A and S3A). In a subset of metaphases showing extensive damage and widespread under condensation conditions, mitotic synthesis foci joined chromosome fragments, overlapping with regions of under-condensation. Consistent with the severity of cytogenetic phenotypes, mitotic synthesis was more frequent in HCT116 than it was in RPE1 cells: mean number of foci per metaphase was 23.2 and 1.53, respectively (Mann- Whitney U test p < 2.2 × 10^−16^). Mitotic synthesis was very frequently associated with cytogenetically visible lesions, especially in the HCT116 cell line (91% of EdU foci coincided with lesions; Figure 3A), suggesting that MIDAS preferentially occurs in the context of cytologically under-condensed mitotic chromatin. We also examined the concurrence between molecular-scale misfolding and mitotic synthesis at the FRA4F site by combining FISH with MIDAS labeling. At that site, MIDAS never appeared on cytogenetically normal regions of the chromosome, even if chromatin at the site showed molecular-scale disruptions indicated by an abnormal FISH signal (Figure 3B). Strikingly, this is similar to observations of MIDAS at telomeres, in which the fragile telomere phenotype did not correlate with the appearance of MIDAS foci (Özer et al., 2018). This observation suggests that, unlike cytogenetic disruptions, molecular-level misfolding is not accompanied by MIDAS and raises the possibility that the misfolding phenotypes represent structures that are independent of DNA replication. To assess whether ongoing DNA synthesis was required for the appearance of classic CFS cytogenetic defects, cells were treated with a high dose of APH during mitosis. The frequency of cytogenetic lesions did not change, indicating that the mitotic-condensation defects are not caused by MIDAS (Figure S3B).

Although the structures underlying both cytogenetic and molecular lesions are unclear, we examined whether they represent intermediates that can be resolved or are permanent defects in mitotic chromatin structure. The duration of G2 after induction of replication stress was artificially prolonged with the CDK1 inhibitor RO3306 to enable aberrant chromatin structures to be resolved before releasing cells to go into mitosis (Figure 3C). The frequency of both cytological lesions and molecular misfolding was significantly reduced after RO3306 treatment, indicating that the structures underlying these phenotypes can be subject to replication and/or repair during G2.

### Chromatin at CFS Regions Is Not Remodeled for Mitosis

**Figure 4:**
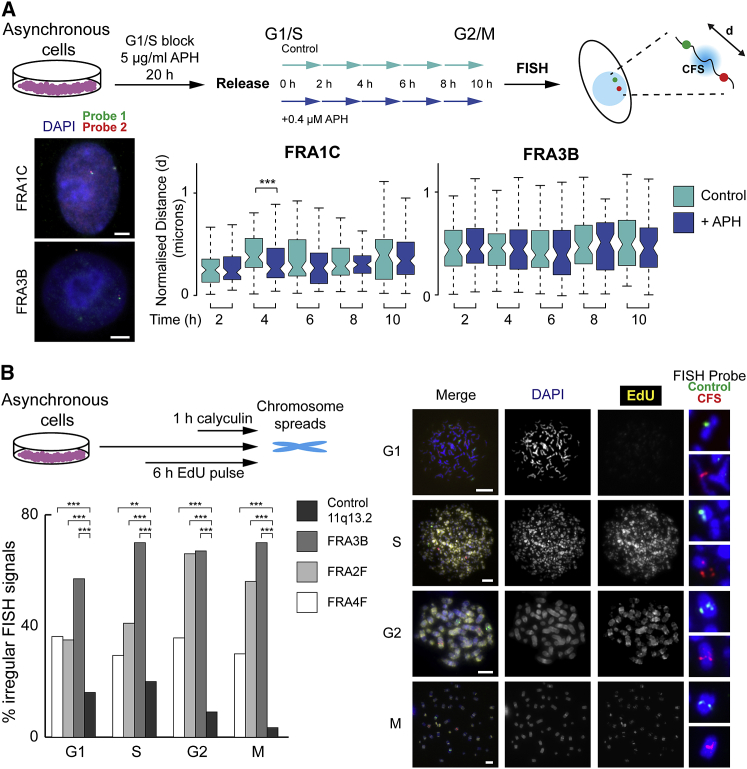
Molecular Chromatin Disruptions at CFSs Are Not Remodeled for Mitosis (A) Top, model detailing experimental strategy to analyze interphase chromatin folding at CFS regions. Cells progressing synchronously through the cell cycle were harvested every 2 h and fosmid probes approximately 1.5 Mb apart surrounding FRA1C (probes B3 and C1) or FRA3B (probes E4 and F5) were hybridized to nuclei and imaged. Bottom left, representative FISH images. Bottom right, boxplot of normalized inter-fosmid distances (d in drawing) between pairs of probes hybridized to nuclei (n > 60 nuclei for each time point). p values are for a Wilcoxon test. (B) Depiction of premature chromosome condensation (PCC) assay (see Method Details) in HCT116 cells. Cells labeled with EdU (6 h) were condensed with calyculin (1 h), harvested, and hybridized to FISH probes for a control locus (11q13.2, probe P21) and CFSs (FRA3B, probe C2; FRA4F, probe A17; and FRA2F, probe K5). Right, representative chromosome images. Bottom, left, quantification of irregular FISH probe signals at FRA3B (n = 122 chromosomes), FRA2F (n = 136), FRA4F (n = 119), and 11q13.2 control probe (n = 116); p values for a χ^2^ test. Scale bars, 2.5 μm. NS, not significant; ^∗^p < 0.05; ^∗∗^p < 0.001; ^∗∗∗^p < 0.001; ^∗∗∗∗^p < 0.0001. See also Figure S4 and Table S2.

Because the mechanism(s) giving rise to aberrant chromatin compaction observed at metaphase (Figures 1A and 2B) were unclear, we investigated the possibility that they might arise as interphase chromatin defects. To test that, a two-probe FISH approach was used to examine interphase chromatin compaction at two fragile sites: FRA3B (HCT116 cells) and FRA1C (RPE1 cells), in the presence and absence of APH (Figure 4A) in synchronized cell populations at different time points throughout the transition from the G1/S boundary through to G2 and mitosis (Figures 4A and S4A). For each site, two differentially labeled probes, encompassing a 1.5-Mb region, were hybridized, and the physical distance between the probes, indicative of the underlying chromatin structure, was measured. No replication-stress-induced changes in interphase chromatin structure were observed in FRA3B and FRA1C after replication, but there was a change in compaction in FRA1C coincident with when the locus replicated in early to mid S phase. These data indicated that replication-stress, per se, does not induce interphase chromatin structural changes that could explain mitotic condensation failure.

Compaction for mitosis involves many compositional and structural changes, which are required to prepare chromatin for condensation, so we speculated that this process was disrupted at CFS regions because of the presence of unreplicated DNA or post-replicative intermediates. To assess the frequency of misfolding lesions at CFS loci throughout the cell cycle, a premature chromosome condensation (PCC) assay was used at three CFSs and a control, non-fragile region on HSA 11q13.2. Cells were treated with the phosphatase inhibitor calyculin A, which triggers chromosome condensation, irrespective of cell-cycle stage (Figure 4B). This resulted in the formation of prematurely condensed chromosomes with morphologies that are indicative of the cell-cycle stage they are derived from: thin and zigzag shaped in G1, fragmented chromatin in S-phase, cross-shaped chromosomes with fuzzy boundaries in G2 cells, and typical metaphase chromosomes in mitotic cells (El Achkar et al., 2005; Ono et al., 2013). To assess the condensation capacity of FRA4F, FRA2I, FRA3B, and the control location at different phases of the cell cycle, FISH probes mapping to the three locations were hybridized, and the morphology of the FISH signals were scored for the different cell-cycle stages (G1, S, G2, and M) in the absence of replication stress. To verify the accuracy of our approach, we quantified the frequencies of one-spot (unreplicated) and two-spot (replicated) signals throughout the different cell-cycle stages and found that, as expected, one-spot signals were more common at the early stages of the cell cycle, and two-spot signals were more frequent in G2 and mitotic chromosomes, especially at the control location (Figure S4B). The analysis revealed contrasting dynamics in chromatin competence for condensation at the CFS sites and the control region (Figure 4B). At the control locus, the frequency of atypical signals decreased in the later phases of the cell cycle, with only a small proportion of signals retaining the misfolded phenotype in G2 and M chromosomes. This indicated that chromatin at the locus acquires competency for mitotic compaction as the cell cycle proceeds. In contrast, at the three CFSs, the atypical FISH signals persisted throughout the cell cycle and remained high in mitotic chromosomes, indicating that the process that allows genomic locations to remodel their chromatin environment and compact for mitosis may be disrupted at CFSs. These results also indicated that the molecular lesions manifested at CFSs in mitosis (Figure 2B) are initiated at earlier cell-cycle stages. To examine condensation dynamics in the context of replication stress, we also induced PCC in cells treated with APH. Although chromosome morphologies indicative of G1 and S could still be distinguished in those cells, post-replicative chromosomes from G2 and mitotic populations could not be distinguished as separate morphologies, instead appearing as G2 chromosomes displaying high levels of fragmentation (Figure S4C). We found that both the control and the FRA4F locus showed high levels of misfolding in post-replicative chromosomes in the presence of APH, suggesting that replication stress, combined with premature condensation, can induce compaction failure, even at non-fragile locations, indicating that the timing of both replication and condensation is important for successful mitotic folding. Additionally, in the presence of replication stress, high levels of mis-folding were observed at the FRA4F region even at G1, possibly because of exposure to APH during the S phase in the previous cell cycle.

### Cytologically Observed Defective Condensin Loading at CFS Regions

**Figure 5:**
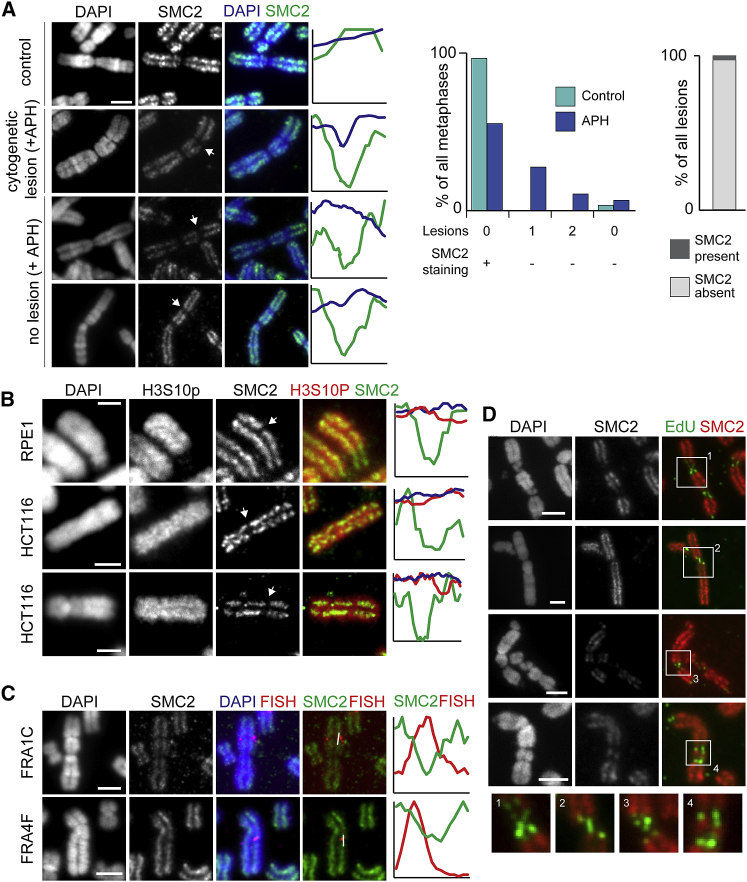
SMC2 Depletion at CFSs (A) Representative images of mitotic chromosomes from RPE1 cells with SMC2 staining in control, untreated, cells (top) and cells treated with APH showing regions of cytogenetic lesions (middle) and on cytogenetically normal chromosomes (bottom) with intensity profiles of DAPI and SMC2 (regions of interest are marked in Figure S5A). Scale bars, 2.5 μm. Middle, frequency of cytogenetic lesions and lesion-free SMC2 depletion in the presence or absence of APH (n = 59 and 76 metaphases for control and APH conditions, respectively). Right, quantification of SMC2 occupancy at cytogenetic lesions (n = 46 lesions). (B) Representative immunofluorescence staining in RPE1 and HCT116 cells for SMC2 and H3S10 phosphorylation on mitotic chromosomes after replication stress, showing regions of SMC2 depletion (arrows). Right, intensity profiles across regions of interest indicated by white line in Figure S5C. Scale bars, 2.5 μm. (C) Representative images showing immunofluorescence staining for SMC2 and FISH for CFSs after replication stress. FISH probes for the FRA1C (probe A14; RPE1 cells, top) or FRA4F (probe A17; HCT116 cells, bottom) sites overlap with regions of SMC2 depletion on metaphase chromosomes. Right, intensity profiles across the region of interest marked by white line. Scale bars, 2.5 μm. (D) Representative images of EdU incorporation marking MIDAS on chromosomes from HCT116 cells co-stained for SMC2. Scale bars, 2.5 μm. Inset shows enlarged area marked by white box. See also Figure S5 and Table S2.

Because we observed that CFSs coincide with large-scale defects in mitotic chromosome topology, we considered defects in condensin-mediated compaction as a potential mechanism that may affect mitotic compaction at CFS loci. Previous work in yeast has indicated that failure to detect post-replicative defects in slow replication zones leads to break formation in a condensin-dependent manner (Hashash et al., 2012). In mammalian cells, condensin recruitment is coupled to replication and abrogated after DNA damage (Zhang et al., 2016). To determine whether similar processes are applicable here, we examined condensin localized to cytogenetic breaks at CFS loci. Using an antibody against SMC2, a component of both condensin complexes in mammalian cells, CFS cytogenetic lesions were frequently found to be depleted of condensin (Figures 5A, S5A, and S5B). Furthermore, the region of condensin depletion appeared to encompass a larger area than the cytogenetic break. On a very small proportion of chromosomes in both the control and APH-treated samples, large regions of SMC2 depletion could be observed in the absence of a cytogenetic break and at cytogenetic locations that were consistent with frequent CFSs, such as FRA1C (Figures 5A and S5B). Co-staining with an antibody targeting the H3 serine 10 phosphorylation mark, which is acquired on chromatin in preparation for mitotic folding, showed that large depletions of SMC2 could be observed in areas that showed high levels of H3S10p signal, indicating that the drop-off in SMC2 signal is not due to reduced DNA content at those regions (Figures 5B and S5C). To verify that regions of SMC2 depletion in the absence of cytogenetic abnormalities occur at CFSs, SMC2 immuno-fluorescence was combined with FISH with probes for the FRA1C and FRA4F CFS regions, which confirmed that SMC2 depletions occur at CFS regions (Figures 5C and S5D). Condensin depletion appeared to be relevant for repair processes at CFSs; MIDAS labeling with SMC2 immunostaining revealed that DNA synthesis only occurs in regions of mitotic chromosomes depleted of SMC2 and showing cytological defects, raising the possibility that uncondensed chromatin, lacking condensin, may be a necessary condition for MIDAS (Figure 5D).

Because condensin phosphorylation by Cdk1 (Abe et al., 2011) and Chk2 (Zhang et al., 2016) is necessary for chromosome compaction, we speculated that failure of condensin loading at CFSs could be triggered by ATM (ataxia-telangiectasia, mutated) or ATR (ATM and Rad3-related) signaling, particularly because inhibition of the Chk2 kinase after DNA damage restores SMC2 association with mitotic chromosomes (Zhang et al., 2016). ATM inhibition in the presence of replication stress caused an increase in cytogenetic lesions per metaphase chromosome and an increase in MIDAS, indicative of a role for ATM in registering damage (Figure S5E). However, SMC2 lesions were still present, both at cytogenetic breaks and on cytogenetically normal chromosomes, suggesting that the ATM-Chk2 pathway was not responsible for blocking condensin loading after replication stress.

Because ATR has a critical role in maintaining replication-dependent genome stability (Cimprich and Cortez, 2008; Saldivar et al., 2018; Zeman and Cimprich, 2014) we also analyzed chromosome architecture after replication stress in the presence of an ATR inhibitor. Consistent with previous studies (Casper et al., 2002; Durkin et al., 2006), inhibition of the ATR-Chk1 axis caused widespread chromosome shattering, and immunostaining indicated SMC2 was not correctly recruited to the chromosomal fragments (Figure S5F). Because ATR has a role in ensuring coordination between the S phase and mitosis (Saldivar et al., 2018), it is likely that chromosome shattering results from premature compaction of under-replicated chromosomes on a genome-wide scale, indicating that condensin cannot be recruited to under-replicated chromatin.

### Condensin I Depletion Causes Mitotic Folding Defects at Non-fragile Locations

Because local condensin depletion correlates to CFS misfolding in mitosis (Figure 5), we sought to examine the effects of global depletion of the condensin complexes. Initially condensin loss was examined in an HCT116 cell line in which both copies of SMC2 were fused to an auxin-inducible degradation (AID) tag (Figure S6A). The HCT116-SMC2-AID cell line showed severe defects in mitotic chromosome structure upon SMC2 degradation: individual chromosomes could not readily be distinguished, and metaphases appeared as a mass of condensed fragments, as described previously (Green et al., 2012). However, MIDAS foci could still be observed in SMC2-depleted metaphases, although at reduced levels (Figure S6B), whereas FISH showed an increase in molecular misfolding at both control and fragile sites (Figure S6C). These results indicated that the absence of the condensin complexes can cause molecular misfolding.

**Figure 6:**
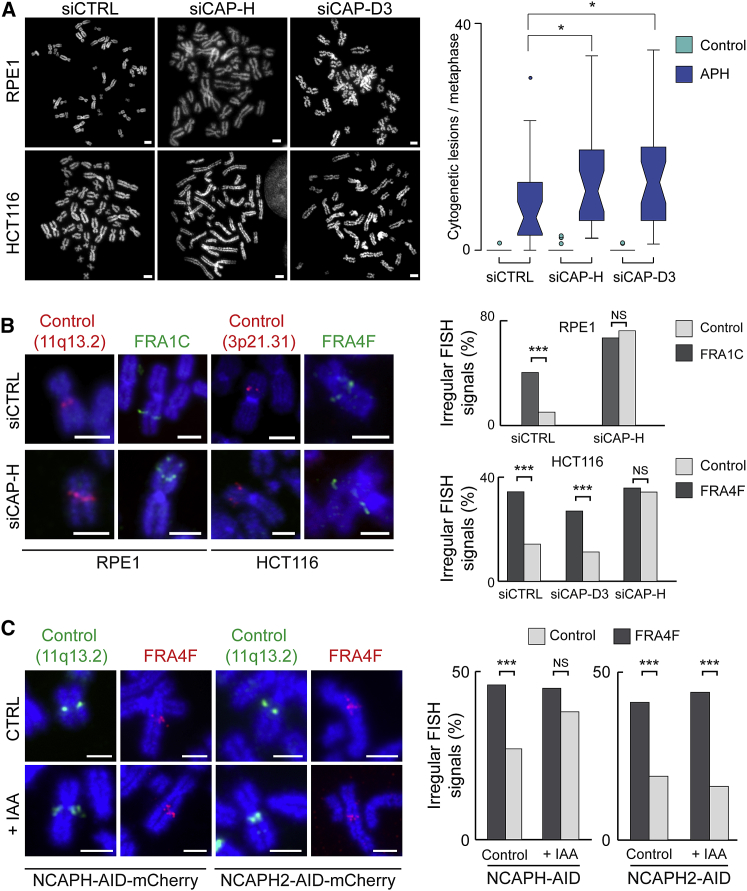
Condensin I Depletion Causes Molecular Chromatin Lesions in Mitosis (A) Left, representative images of chromosomal defects in the HCT116 and RPE1 cell lines after siRNA depletion of condensin components CAP-H (condensin I) or CAP-D3 (condensin II) compared with siRNA control (siCTRL). Scale bars, 2.5 μm. Right, quantification of cytogenetic lesions per metaphase after condensin depletion in the HCT116 cell line in the absence (green) or presence (blue) of APH (n > 20 metaphases per condition). p values are for Student’s t test. (B) Left, representative images of chromosomal defects visualized by FISH at FRA1C (probe A14) and FRA4F (probe A17) fragile sites and control loci (11q13.2, probe P21; 3p21.31, probe C14) in RPE1 and HCT116 cell lines after depletion of the condensin component CAP-H. Scale bars, 5 μm. Right, quantification of abnormal FISH signals, indicative of chromatin disruptions, per metaphase after condensin depletion in the HCT116 and RPE1 cell lines (n > 40 for each condition). p values are for a χ^2^ test. (C) Left, representative images showing FISH probes at the fragile FRA4F (probe A17) region and at a non-fragile control region (11q13.2, probe P21) after degradation of the condensin I component CAP-H in HCT116 cells. Scale bars, 2.5 μm. Right, quantification of the frequency of irregular FISH signals, indicative of CAP-H-dependent mitotic chromosome misfolding (n > 100 chromosomes per condition). p values are for a χ^2^ test. NS, not significant; ^∗^p < 0.05, ^∗∗^p < 0.001, ^∗∗∗^p < 0.001, ^∗∗∗∗^p < 0.0001. See also Figure S6 and Table S2.

To explore the different roles of the condensin complexes, small interfering RNAs (siRNAs) against CAP-H and CAP-D3 were used to deplete condensin I and condensin II complexes, respectively (Figure S6D). As for SMC2 depletion (Figure S6B), depletion of either condensin complex resulted in defects in mitotic chromosome morphology (Green et al., 2012); condensin-II-depleted chromosomes had a pronounced wavy appearance (Figure 6A). Scoring of cytogenetic lesions (Figure 6A) and MIDAS foci (Figure S6E) in those chromosomes revealed that, although CAP-H or CAP-D3 depletion did not induce cytogenetic lesion formation in unperturbed conditions, there was a significant increase in the frequency of CFS cytogenetic lesions in condensin-depleted chromosomes and MIDAS once replication stress was induced. This is indicative of both replication stress and aberrant condensin exerting additive effects on cytogenetic lesion formation at CFS.

We next examined the effect of CAP-H or CAP-D3 siRNA depletion on the mitotic misfolding phenotype at CFS and control, non-fragile locations by FISH, in the absence of replication stress. The frequency of misfolding at CFS locations was significantly greater than control loci in cells treated with scrambled siRNA (siCTRL) in both the RPE1 and HCT116 cell line (Figure 6B). In cells depleted of CAP-H, the frequency of misfolding was unaltered at CFSs but increased significantly at control loci to levels that matched CFSs, suggesting that depletion of condensin I is sufficient to recapitulate the misfolding phenotype characteristic of CFS sites at a non-fragile location. In contrast, depletion of CAP-D3 (condensin II) did not affect the frequency of misfolding at non-fragile locations, indicating that the condensin I complex is the primary effector of mitotic misfolding at CFS locations. Additionally, the frequency of misfolding, observed at the control locus was similar after SMC2 depletion (46%; Figure S6B) and CAP-H depletion (34%; Figure 6B), suggesting that the effect of SMC2 depletion is explained by removing condensin I from chromosomes.

To further investigate the role of condensin using an orthogonal approach, we used HCT116 cell lines in which both copies of CAP-H or CAP-H2 were fused to an AID tag, which enabled rapid depletion of either condensin I or II upon addition of auxin (indole-3-accetic acid [IAA]) for 8 h (Figure S6F) (Takagi et al., 2018). Mitotic misfolding in the absence of replication stress, measured by irregular FISH signals, for a control locus at 11q13.2 and the FRA4F fragile site were analyzed before and after auxin treatment. As already observed at those sites (Figure 6B), the fragile site locus had a greater extent of mitotic chromosome misfolding in the absence of condensin degradation compared with the control. Although the levels of misfolding remained unchanged at the CFS after the degradation of CAP-H, increased misfolding was observed for the control locus after CAP-H degradation was triggered (Figure 6C), indicating that defects in condensin I loading are sufficient to induce mitotic misfolding. In contrast, CAP-H2 degradation did not lead to an increase in misfolding at the control locus, confirming that condensin I is the primary effector of mitotic defects at CFS.

## Discussion

**Figure 7:**
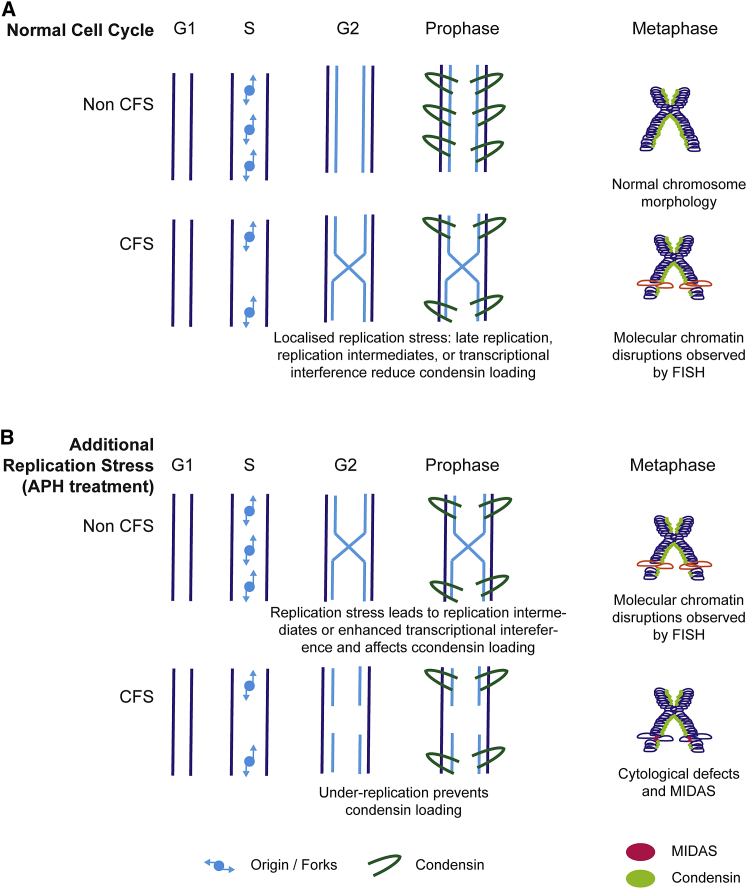
Model Showing the Formation of Chromosome Lesions from Faulty Condensin Loading after Replication Stress (A) Under normal cell cycle conditions control loci are replicated and recruit condensin in prometaphase. Together, condensin I and II activity results in helically arranged, nested loop arrays giving rise to metaphase chromosomes. Although this occurs at most sites across the genome, there are some regions, including common fragile sites, in which the local chromatin environment is refractory to replication. This could occur through transcriptional interference, aberrant DNA resolution, or other unknown processes but affects condensin loading. Loss of condensin results in molecular lesions, visible by FISH, caused by a local inability to package loci into mitotic chromosomes. (B) APH treatment creates additional replication stress. At typical chromosomal loci, this may result in replication intermediates or enhanced transcriptional interference that reduces condensin loading, leading to chromatin disruptions that can be observed by FISH. At CFSs, this phenomenon is more extreme, resulting in under-DNA replication, which prevents condensin loading. After prometaphase cytological lesions are coincident with MIDAS, extending G2 provides time for replication to complete suppressing chromosome lesions and irregular FISH signals at CFSs.

Replication stress affects genome-wide alterations in fork behavior, leading to activation of extra origins, changes in origin efficiency, and potentially, altered replication dynamics (Courbet et al., 2008; Macheret and Halazonetis, 2018). A number of factors can trigger replication stress: oncogene activation, misincorporation of nucleotides, or replication-transcription conflicts (Helmrich et al., 2011; Hills and Diffley, 2014; Reijns et al., 2015), but an often overlooked aspect of replication stress is the local chromatin environment before and after replication. Pre-replication, DNA supercoiling (Naughton et al., 2013), catenanes, paucity of active chromatin marks, and unusual DNA structures, such as R loops and G-quadruplexes, have all been shown to interfere with replication dynamics, suggesting that features of the underlying chromatin environment could be a critical factor linking replication stress to genome instability (Comoglio et al., 2015). We suggest that replication stress interferes with the setup of the post-replicative chromatin environment (Figure 7). This is most obvious at CFS regions, where under-replication or persisting post-replicative intermediates prevent condensin loading. In unperturbed conditions, this results in subtle mitotic misfolding, specific to CFS regions, which is only visible by FISH. In conditions of replication stress, the severity of condensin-loading defects increases, leading to classic cytogenetic lesions accompanied by MIDAS. Extending G2 allows for structures impeding condensin loading to be resolved, leading to a decrease in both molecular misfolding and cytogenetic lesions. In contrast, inducing premature condensation in the presence of replication stress or preventing condensin loading through condensin depletion induces high levels of misfolding at non-fragile genomic regions. These results hint at the careful coordination between replication and mitotic compaction, which is disrupted at CFS in both unperturbed conditions and after replication stress.

During our analysis of the effect of replication stress on mitotic compaction, we report a new layer of instability at CFSs, visible at the molecular, but not the cytogenetic, level (Figure 2). These aberrant structures bear similarity to phenotypes previously seen at telomeres and at centromeres after replication stress (Sfeir et al., 2009). At telomeres, lesions are thought to result from replication problems, such as fork collapses or G-quadruplex structures formed by GC-rich telomeric repeats; however, CFSs are not composed of repetitive sequences. and it is unclear how small-scale events can lead to fragility and failure of mitotic compaction on such a large genomic scale, suggesting additional factors like chromatin structure, epigenetics, and replication dynamics have a role, as previously proposed (Letessier et al., 2011; Macheret et al., 2020). The similarity between CFS phenotypes supports the idea that mitotic misfolding is a universal feature of CFSs and potentially, other difficult-to-replicate regions. Although classic cytogenetic lesions that characterize CFSs cannot be observed in the absence of APH, this newly characterized low level of instability, apparent using FISH, is present at these loci at a low frequency, even when cells undergo normal replication (Figure 7). It is unclear what the exact relationship is between the aberrant folding observed by FISH and the classic, cytogenetic CFS lesions; however, aberrant folding is necessary but not sufficient for the formation of classical CFS abnormalities. This finding indicates the inherent fragility present at CFS regions even in the absence of exogenous replication stress and implicates a model for their instability in physiological contexts, such as during tumor development (Alexandrov et al., 2013). Surprisingly, our data also show that, after APH treatment, molecular-scale lesions are also present at control loci (Figure 2C), indicating a genome-wide link between replication stress and chromatin compaction.

Although traditional models envision CFS instability as mediated primarily through replication dynamics, we suggest that the aberrant processing of post-replicative chromatin, resulting in disrupted mitotic folding, also has a role. Most genomic regions undergo compositional and structural remodeling, or “priming” of chromatin, which facilitates mitotic condensation and sister chromatin separation. This idea is not without precedent: the Kleckner et al. (2004) group have suggested that, through the cell cycle, chromatin is continuously remodeled using energy stored in the chromatin fiber through tethering, which, when released, allows chromatin expansion (Liang et al., 2015). The mechanistic steps for chromatin folding from interphase to mitosis are poorly defined, but key events include condensin loading, histone H3 phosphorylation, and catenane resolution, by topoisomerases; all of which may influence the potential energy stored within the fiber. Our data indicate that CFSs are inefficiently primed (Figure 4), show faulty condensin recruitment (Figure 5), and remain refractory to compaction (Figure 7).

In addition to an inherent high level of molecular misfolding (Figure 2), MIDAS is a frequent feature of CFSs (Figure 3). Chromosome lesions provide a permissive environment for the MIDAS process, and most synthesis occurs in the context of uncondensed chromatin, which is free of condensin (Figure 5D). Our data suggested that neither condensin I nor condensin II were required for MIDAS (Figures 6B and S6E), in contrast to a previous study (Minocherhomji et al., 2015). However, in the data presented here, SMC2 was depleted with a degron-based system instead of RNAi. This resulted in low levels of SMC2 and a very aberrant chromosome morphology. It has been previously proposed that the MIDAS process constitutes a repair pathway for lesions before the completion of mitosis (Minocherhomji et al., 2015; Naim et al., 2013), but our observations suggest that altered chromatin compaction at CFS could also aid their repair. Remarkably, under-condensation in mitosis at unresolved homologous recombination intermediates was found to aid cell division, although those mitotic structures represented a distinct phenotype from CFS lesions (Chan et al., 2018). The primary causes of impaired compaction at CFSs are complex: our data suggest that under-replication, repair intermediates, or aberrant chromatin structures resulting from them impair condensin recruitment. Consistently extending G2 to allow for the repair of intermediates results in a reduction of both cytogenetic abnormalities and mitotic misfolding (Figure 3C).

Chromosome misfolding is not restricted to CFSs because normal genomic regions show a low frequency of lesion formation in the presence of replication stress, suggesting that common fragile sites do not have a unique set of chromatin features (Figure 2). Instead, CFSs are at the extreme end of a spectrum of aberrant chromatin structures that have a propensity to exhibit replication stress and inefficient priming leading to misfolding in mitosis and subsequent chromosome instability if not repaired. Counterintuitively, cytological lesions may provide a chromosomal environment to facilitate processes such as MIDAS to repair chromatin.

## STAR★Methods

### Key Resources Table

**Table:** 

REAGENT or RESOURCE	SOURCE	IDENTIFIER
**Antibodies**		

CAP-H	Bethyl	Cat# A300-603A; RRID:AB_2150006
CAP-D3	Bethyl	Cat# A300-604A; RRID:AB_2298269
GAPDH	Cell Signaling	Cat# 2118; RRID:AB_561053
H3S10pclone CMA313	Gift from Hiroshi Kimura	N/A
Rabbit anti-SMC2	Kumiko Samejima	N/A
Texas Red anti-mouse	Jackson Immuno Research	Cat# 715-585-150; RRID:AB_2340854
FITC anti-rabbit	Jackson Immuno Research	Cat# 711-095-152; RRID:AB_2315776
FITC avidin	Vector Labs	Cat# A-2011; RRID:AB_2336456
Biotin anti-avidin	Vector Labs	Cat# BA-0300; RRID:AB_2336108
Texas Red anti-sheep	Vector Labs	Cat# TI-6000; RRID:AB_2336219
Anti-dig rhodamine	Roche	Cat# 11207750910; RRID:AB_514501

**Chemicals, Peptides, and Recombinant Proteins**		

Aphidicolin	Calbiochem	38966-21-1
Colcemid	Life Technologies	15210-040
Calyculin	Simga	C5552-10UG
biotin-16-dUTP	Roche	11093070910
digoxigenin-11-dUTP	Roche	11093088910
Alexa Fluor 488 Azide	Thermo Fisher	A10266
DNA Polymerase I	Invitrogen	18010025
RNase A	Invitrogen	12091039
DNaseI	Roche	4716728001

**Critical Commercial Assays**		

Click-it Plus EdU Alexa Fluor 647 Flow Cytometry Assay Kit	Invitrogen	C10634

**Experimental Models: Cell Lines**		

hTERT RPE1	ATCC	CRL-4000
HCT 116	ATCC	CCL-247
SMC2-AID-mClover HCT 116	This study	N/A
CAPH-AID-mCherry HCT 116	Takagi et al.	N/A
CAPH2-AID-mCherry HCT 116	Takagi et al.	N/A

**Recombinant DNA**		

BAC: RP11-357C16	BACPAC Resources	N/A
BAC: RP11-452B11	BACPAC Resources	N/A
BAC: RP11-482A14	BACPAC Resources	N/A
BAC: RP11-44E15	BACPAC Resources	N/A
BAC: RP11-624N7	BACPAC Resources	N/A
Fosmid: G248P86197B3	BACPAC Resources	N/A
Fosmid: G248P83504C1	BACPAC Resources	N/A
BAC: RP11-436I1	BACPAC Resources	N/A
BAC: RP11-236P10	BACPAC Resources	N/A
BAC: RP11-56K5	BACPAC Resources	N/A
Fosmid: G248P8183F5	BACPAC Resources	N/A
Fosmid: G248P89337E4	BACPAC Resources	N/A
BAC: RP11-27C2	BACPAC Resources	N/A
BAC: RP11-1053C2	BACPAC Resources	N/A
BAC: RP11-44A17	BACPAC Resources	N/A
BAC: RP11-351L22	BACPAC Resources	N/A
BAC: RP11-479E18	BACPAC Resources	N/A
BAC: RP11-915N9	BACPAC Resources	N/A
BAC: RP11-6L24	BACPAC Resources	N/A
BAC: RP11-946L12	BACPAC Resources	N/A
BAC: RP11-153D1	BACPAC Resources	N/A
BAC: RP11-126P21	BACPAC Resources	N/A
BAC: RP11-795A13	BACPAC Resources	N/A
BAC: RP11-412C14	BACPAC Resources	N/A

**Software and Algorithms**		

ImageJ	Open Source	N/A

### Resource Availability

#### Lead Contact

Further information and requests for resources and reagents should be directed to the Lead Contact, Nick Gilbert (nick.gilbert@ed.ac.uk).

#### Materials Availability

All unique/stable reagents generated in this study are available from the Lead Contact without restriction.

This study generated SMC2-AID HCT116 cells.

#### Data and Code Availability

This study did not generate any large-scale datasets.

### Experimental Model and Subject Details

RPE1 (female) and HCT116 (male) cells were cultured in Dulbecco’s Modified Eagle Medium F12 (GIBCO, Cat No. 12500-062), supplemented with 10% fetal calf serum, 1% Pen-Strep and 1% L-glutamine. Additionally, growth media for RPE cells also contained 0.3% (w/v) Sodium Bicarbonate (Sigma, Cat. No. S5761). All cells were maintained at 37 °C in an atmosphere of 5% CO2. HCT116 degron cell lines were grown in McCoy’s 5A medium (GIBCO, Cat No. 26600-023) supplemented with 10% fetal calf serum and 3 mM L-glutamine. All cell lines were subjected to regular mycoplasma testing. Cell authentication was performed via karyotyping.

### Method Details

#### Cell culture transfections

Vectors were transfected into cells using Lipofectamine 2000 (Invitrogen, Cat. No. 11668-019) and Opti-MEM Reduced Serum Medium (Invitrogen, Cat. No. 31985-070). For each transfection 1 μg of construct DNA was mixed with 400 μL Opti-Mem and 5 μL Lipofectamine-2000. To avoid aggregation of DNA and Lipofectamine-2000, the DNA was pre-mixed in 200 μL of Opti-Mem and separately, the 5 μL of Lipofectamine were mixed into 200 μL of Opti-mem. The two components were then mixed together and incubated for 20 min at RT. This transfection mixture was added to the tissue cultures in 2 mL of antibiotic-free media.

#### Protein gels and western blotting

Cells were suspended in NuPAGE LDS sample buffer (ThermoFisher) with 10 mM DTT, incubated at 100°C for 5 min and sonicated briefly. Protein samples were resolved on 8% bis-tris gels (ThermoFisher) and transferred to Immobilon-P PVDF 0.45 μm membrane (Merck Millipore) by wet transfer. Membranes were probed with antibodies using standard techniques and detected by enhanced chemiluminescence. Antibodies used for western blotting were as follows: CAP-H (Bethyl A300-603A, 1:1000), CAP-D3 (Bethyl, A300-604A, 1:1000) and GAPDH (Cell Signaling 2118L, 1:5000).

#### Cell cycle synchronization and replication stress induction

Cells were synchronized at the G1/S boundary by addition of high dose aphidicolin (APH, Calbiochem). Media containing 5 μg/ml APH was added to cells for 2 h to block cell cycle and retain cells at the G1/S boundary. Cells were washed in PBS and released in normal growth media. FACS analysis and immunofluorescence of cell populations at 2 h – 10 h following release showed that cells progressed synchronously from S-phase into G2. Replication stress was induced by low dose treatment of APH (0.4 μM APH), for extended periods (12 – 24 h).

#### Preparation of human metaphase chromosomes

RPE1 cells were treated with 0.1 μg/ ml colcemid (Life Technologies, Cat No 15210-040) for 1 h prior to harvest, and HCT116 for 30 min to induce mitotic arrest and increase the number of mitotic cells. Cells were trypsinised and washed in PBS. Hypotonic solution, containing 75 mM KCl was added drop wise to a final 5 mL volume. Hypotonic treatment was performed at RT for 10 min, after which cells were pelleted by centrifugation at 1200 rpm for 5 min and fixed three times in 5 mL of freshly prepared solution of 3:1 ratio (v/v) methanol: acetic acid. The MAA fixative was added to the cell pellet dropwise with constant agitation. Chromosome preparations were stored at −20°C. To prepare slides with metaphase spreads, metaphase chromosome preparations were dropped onto glass slides. The glass slides were pre-treated in a dilute solution of HCl in ethanol for at least an hour prior to use. The chromosome preparations were pelleted by centrifugation at 1500 rpm for 5 min and resuspended in freshly prepared MAA solution until the suspension became cloudy. Two drops of the suspension were dropped onto a pre-treated glass slide from a height of 20 cm and dried at RT overnight before staining or hybridization.

#### Cytogenetic analysis of common fragile sites

To map the location of fragile sites two complementary approaches were used. First, a visual inference of the fragile locus position was made using reverse DAPI banding. Second, the position of the fragile site was determined by calculating the distance along the chromosome arm. In this ratio-based approach, the total length (a), in pixels, of the chromosome arm that the break occurred on and the pixel length of the distance between the centromere and the break (b) were measured. The ratio (b) / (a) was calculated and used on scaled models of banded chromosomes (from the International System for Human Cytogenetic Nomenclature) to infer genomic locations for the breaks. The ratios clustered along the chromosome arms, indicating recurrent breaks at CFS locations and the mid-point of each cluster was taken as a putative CFS location. However, as fixation and spreading of chromosomes is likely to cause some distortion, molecular fine-mapping of the most frequent CFS regions was also undertaken using FISH.

#### Fluorescence *in situ* hybridization (FISH)

Probes used in this study are listed in Table S2. After mapping fragile sites, the following probes were used to interrogate genomic loci by FISH (probe ID: A14, A17, K5, C2, L12, A13, P21, C14). DNA was prepared from the BACs or Fosmids and labeled as previously described (Naughton et al., 2010). Probes were labeled using a nick translation reaction with the uridine analogs biotin-16-dUTP (Roche, CatNo 11093070910) or digoxigenin-11-dUTP (Roche, CatNo 11093088910). Nick translation was performed in a 20 μL reaction volume, containing 1-1.5 μg DNA with 5 μL each of 0.5 mM dATP, dCTP and dGTP and either 2.5 μL of 1 mM biotin-16-dUTP or 1 μL of 1 mM digoxigenin-11-dUTP. DNase I (Roche, Cat No 4716728001) was added to a final concentration of 1 U/ml and DNA polymerase I (Invitrogen, Cat No 18010025) was added to a final concentration 0.5 U/μl. The reaction was performed in 1 x nick translation salts (NTS) buffer, containing 50 mM Tris pH7.5, 10 mM MgSO4, 0.1 mM DTT and 50 μg/ml BSA for 90 min at 16°C. Unincorporated nucleotides were removed by gel filtration of the NTS reaction through a G50 Sephadex spin column (Roche, Cat No G50DNA-RO). Slides, containing either MAA-fixed chromosome spreads or PFA-fixed nuclei, were treated with 100 μg/ml RNaseA (Invitrogen, Cat No 12091039) in 2 x SSC for 1 h at 37°C, washed briefly in 2 x SSC and dehydrated through an ethanol series (2 min each in 70%, 90% and 100% ethanol). Slides were air-dried and baked at 70°C for five min before denaturing. Denaturation was performed in 70% formamide (v/v) in 2 x SSC (pH 7.5). Slides containing MAA-fixed chromosome spreads were denatured at 70°C for 1 min, while slides on which cells were cultured and then fixed in 4% PFA were denatured at 80°C for 20 min. Following denaturation, slides were submerged in ice-cold 70% ethanol for 2 min and then dehydrated through 90% and 100% ethanol for 2 min each at RT. For hybridization, 150 ng of labeled probe was combined with 5 μg of salmon sperm and 10 μg of human Cot1 DNA (Invitrogen, Cat No 15279011). Two volumes of ethanol were added and the probe mix was collected by centrifugation and dried. Dried probes were resuspended in 10 μL of hybridization buffer containing 50% formamide (v/v), 1% Tween-20 and 10% dextran sulfate (Sigma Aldrich, Cat No D8906-100G) in 2 x SSC. Probes were denatured at 70°C for 5 min and reannealed at 37°C for 15 min and chilled on ice. Probes were pipetted onto slides and hybridization was performed at 37°C overnight. Coverslips were then removed and slides were washed four times in 2 x SSC at 45°C for 3 min and four times in 0.1 x SSC at 60°C for 3 min. Slides were then blocked in 5% milk in 4 x SSC for 5 min at RT. Detection of biotin label was performed with sequential layers of fluorescein (FITC)-conjugated avidin, biotinylated anti-avidin and a further layer of FITC-avidin. Digoxigenin was detected with sequential layers of Rhodamine-conjugated anti-digoxigenin and Texas-Red (TR) –conjugated anti-sheep IgG. Slides were DAPI stained, mounted in Vectashield and imaged on a Zeiss epifluorescence microscope using a 100x objective. Data was collected using micromanager software and analyzed using custom scripts in iVision or ImageJ. FISH signals were scored based on symmetry and overlap with DAPI chromosome staining: FISH probes showing asymmetric signals or signals extending outside the chromosome scaffold were classified as irregular, while symmetric signals contained within the chromosome scaffold were classified as regular. Whenever possible, scoring was performed blindly to experimental conditions. In FISH experiments measuring inter-probe distance, ambiguous images in which the two alleles could not be distinguished, were not used in the analysis.

#### Premature chromosome condensation (PCC) assay

Premature chromosome condensation was induced using the protein phosphatase 1 inhibitor calyculin (Sigma, C5552-10UG). To determine the cell cycle stage of prematurely compacted chromosomes, asynchronously growing HCT116 cells were pulsed with EdU (5 μM) for 6 hours and then treated with 50 ng/ml calyculin for 1 hour. Cells were then harvested using trypsin/versene. As calyculin treatment induced significant cell detachment, media containing the detached cells was collected and centrifuged together with the trypsinised cells. Metaphase spreads were prepared and dropped onto glass slides using the methods described above. Incorporated EdU was labeled in a click reaction by incubating slides with a reaction mixture containing 500 μg/ml CuSO4, 40 μM Alexa Fluor 488 Azide (Thermo Fisher Cat. No. A10266) and 20 mg/ml ascorbic acid for 1 hour at RT. Slides were washed three times in PBS for 5 min, stained with DAPI and mounted in Vectashield. Slides were imaged on a Zeiss Epifluorescence microscope using 100x objective. Chromosomal morphology and EdU staining pattern were used to classify chromosomes into G1, S, G2 and M-stage chromosomes.

#### Immunofluorescence

For immunofluorescence on metaphase chromosomes, cell suspensions fixed in 3:1 methanol: acetic acid were dropped onto glass slides, allowed to dry incompletely and immediately immersed in PBS for 5 minutes at room temperature. Slides were washed in TEEN buffer (10 mM Triethanolamine- HCl pH 8.5, 2 mM EDTA, 250 mM NaCl) and blocked in 10% fetal calf serum (FCS) at 37°C for 10 minutes. Primary antibodies were added at the required dilutions and incubated in a humidified chamber at 37°C for 30 minutes. Slides were then washed in KB buffer (100 mM Tris-HCl pH 7.7, 1.5 M NaCl, 1% BSA). Secondary antibodies, raised in donkey and conjugated to fluorophores (Jackson Immuno Research), were diluted 1:500 in TEEN buffer, added to the slides and incubated at 37°C for 30 minutes. Slides were washed in KB buffer and stained in 50 μg / ml DAPI for 3 min at RT to detect DNA and nuclei. Slides were mounted in Vectashield (Vector Laboratories, Cat No H-1000) and imaged on a Zeiss Epifluorescence microscope using 100x objective. Primary antibody anti-H3S10p (1:100 dilution, clone CMA313) was a gift from Hiroshi Kimura (Hayashi-Takanaka et al., 2009), and detected with a Texas Red anti-mouse secondary antibody (1:500 dilution, Jackson Immuno Research). Anti-SMC2 antibody was detected with an FITC labebelled anti-rabbit secondary antibody (1:500 dilution, Jackson Immuno Research).

#### EdU labeling and detection

EdU was added to exponentially growing cell cultures for 30 min at 5 μM for replication labeling or 20 μM for analyzing mitotic DNA synthesis. EdU was detected by incubating slides with a click reaction mixture containing 500 μg/ml CuSO4, 40 μM Alexa Fluor 488 Azide (Thermo Fisher Cat. No. A10266) and 20 mg/ml ascorbic acid for 1 hour at RT. Slides were washed three times in PBS for 5 min, stained with DAPI and mounted in Vectashield. Slides were imaged on a Zeiss Epifluorescence microscope using 100x objective.

#### Flow cytometry

For dual EdU and propidium iodide (PI) staining for cell cycle analysis, cells were trypsinised, pelleted and resuspended in PBS at a density of 1.5 × 10^6^ cells/ml. Ethanol was slowly added to the cell suspension to a concentration of 70% to fix and permeabilise the cells, which were incubated on ice for a minimum of 30 min or stored at 4°C for up to 2 weeks. EdU staining was performed using the Click-iT Plus EdU Alexa Fluor 647 Flow Cytometry Assay Kit (Invitrogen, CatNo C10634) following the manufacturer’s instructions. Cells were stained in a solution containing 1 μg/ml PI and 4 μg/ml RNase A in PBS at 2 × 10^6^ cells/ml for a minimum of 30 min at RT. Cell cycle analysis was performed on a LSR Fortessa analyzer (BD Biosciences) and analyzed using FlowJo software.

#### HCT116 condensin degron cell lines

SMC2-AID-mClover cells were a derivative of tet-OsTIR1 HCT116 cells established in the Kanemaki laboratory (Natsume et al., 2016). C terminus targeting constructs for the SMC2 gene contained a 5′ homology arm (410 bp), mAID tag, mClover, resistance cassette and a 3′ homology arm (482 bp) (mAID tag, mClover tag and Hygromycin or G418 resistance cassettes were taken from pMK289 and pMK290. The guide RNA target sequence was TCCACATGTGCTCCTTTGGG. Constructs and resultant cell lines were established using published approaches from the Kanemaki laboratory (Natsume et al., 2016). CAPH-AID-mCherry and CAPH2-AID-mCherry HCT116 cells were a kind gift from the Imamoto lab, RIKEN, Japan (Takagi et al., 2018). For SMC2 degradation SMC2-AID-clover cells were incubated with 1μg/mL doxycycline overnight to induce OsTir1 expression and treated with 500μM Indole-3-acetic acid (IAA) for 24 h. For CAP-H and CAP-H2 degradation, HCT116 cells expressing AID tagged proteins were incubated with 500 μM Indole-3-acetic acid (IAA) for 8 h.

#### Computational analysis

All genomic coordinates are HG38. COSMIC mutations were assessed at CFSs by examining the COSMIC track on the UCSC browser.

### Quantification and Statistical Analysis

The statistical significance of locus compaction was tested using a nonparametric Mann–Whitney U (Wilcoxon) test (using R programming). Comparisons between normally distributed data were tested for significance using a two-tailed Student t test. A Chi square test was used to determine if there was a statistically significant difference between the expected frequencies and the observed frequencies in one or more categories of a contingency table. p < 0.05 was taken as statistically significant.

## Supplemental Information

Document S1. Figures S1–S6 and Tables S1 and S2

Document S2. Article plus Supplemental Information
