# Supplementary material for: Common Fragile Sites Are Characterized by Faulty Condensin Loading after Replication Stress
Source: Cell Rep. 2020 Sep 22;32(12):108177. doi: 10.1016/j.celrep.2020.108177 (PMC7511797; doi:10.1016/j.celrep.2020.108177)
Supplement: Document S1. Figures S1–S6 and Tables S1 and S2 [file mmc1.pdf]

Cell Reports, Volume *32*

Supplemental Information

Common Fragile Sites Are Characterized by Faulty Condensin Loading after Replication Stress

Lora Boteva, Ryu-Suke Nozawa, Catherine Naughton, Kumiko Samejima, William C. Earnshaw, and Nick Gilbert

Supplementary Table 1. Common fragile sites analysed in HCT116 and RPE1 cell lines using DAPI banding and fine-mapping using FISH probes (co-ordinates hg38). Relates to Figure 1.

CFS Name	Cell type specificity (% of all breaks observed in cell type)		Genomic Location	Finemapping	Replication timing
	RPE1	HCT116			
FRA1C	18.6 %	5.8 %	1p31.2	Fine mapping with BAC probes. Fragility found at a 0.6 Mb region around chr1: 68.7-69.3 Mb	Mid/late
Novel	11.9 %	Not fragile	4q32.2	Fine-mapping with BAC probes. Fragility found to span a 1Mb region overlapping with the MARCH1 gene at 4q32.2-4q32.3 boundary	Late
FRA3B	Not fragile	23.4 %	3p14.2	Fine-mapping with fosmid probes. Fragility localised to a 1 Mb region overlapping with the FHIT gene at 3p14.2	Mid/Late
FRA4F	Not fragile	11.0 %	4q22.2	Fine-mapping with BAC probes. Fragility localised to a 5 Mb region between chr4: 88.3-94.2 Mb	Late
FRA2F	8.5 %	5.8 %	2q22.2	Fine-mapping with BAC probes. Fragility localised to a 2 Mb region between chr2: 141.4-143.3 Mb	Mid
FRA3O	16.9 %	1.3%	3q26.31		Late
FRA7E	10.2%	Not fragile	7q21.11		Mid
FRA2I	Not fragile	17.5 %	2q33.2		Mid/Late
FRA2T	Not fragile	9.7 %	2q24.2		Mid
FRA4C	6.8 %	Not Fragile	4q31.1		Early /Mid
FRA7K	Not Fragile	7.1 %	7q31.1		Mid / Late
Control	Not fragile	Not fragile	11q13.2		Early

Table S2. List of fosmid and BAC probes, relates to Figures 2, 3, 4, 5, 6, S2, S3, S4, S5, S6

Probe	Short Probe ID	Probe location	Genomic Band	Type	CFS
RP11-357C16	C16	chr1:68,449,756-68,639,532	1p31.1	BAC	FRA1C
RP11-452B11	B11	chr1:68,711,269-68,891,160	1p31.1	BAC	FRA1C
RP11-482A14	A14	chr1:68,933,453-69,111,196	1p31.1	BAC	FRA1C
RP11-44E15	E15	chr1:69130083-69315886	1p31.1	BAC	FRA1C
RP11-624N7	N7	chr1:68,111,085-68,283,400	1p31.3	BAC	FRA1C
G248P86197B3	B3	chr1:68350693-68390943	1p31.3	Fosmid	FRA1C
G248P83504C1	C1	chr1:69563598-69605132	1p31.1	Fosmid	FRA1C
RP11-436I1	I1	chr2:136,061,231-136,243,657	2q22.1	BAC	FRA2F
RP11-236P10	P10	chr2:140,424,618-140,580,291	2q22.1	BAC	FRA2F
RP11-56K5	K5	chr2:144248998-144409403	2q22.3	BAC	FRA2F
G248P8183F5	F5	chr3:60989838-61032460	3p14.2	Fosmid	FRA3B
G248P89337E4	E4	chr3:59462385-59498598	3p14.2	Fosmid	FRA3B
RP11-27C2	C2	chr3:60,370,532-60,541,081	3p14.2	BAC	FRA3B
RP11-1053C2	C2	chr4:88,292,015-88,468,493	4q22.1	BAC	FRA4F
RP11-44A17	A17	chr4:90613586-90767458	4q22.1	BAC	FRA4F
RP11-351L22	L22	chr4:91,991,524-92,152,798	4q22.1	BAC	FRA4F
RP11-479E18	E18	chr4:94,041,818-94,200,058	4q22.2 - 4q22.3	BAC	FRA4F
RP11-915N9	N9	chr4:85,542,403-85,705,769	4q22.3	BAC	FRA4F
RP11-6L24	L24	chr4:96,170,141-96,329,507	4q22.3	BAC	FRA4F
RP11-946L12	L12	chr4:163,180,808-163,361,254	4q32.2	BAC	Novel
RP11-153D1	D1	chr4:164799551-164962649	4q32.3	BAC	Novel
RP11-126P21	P21	chr11:67186953-67348953	11q13.2	BAC	Control
RP11-795A13	A13	chr1:69385353-69559490	1q42.3	BAC	Control
RP11-412C14	C14	chr3:47618135-47778557	3p21.31	BAC	Control

Fosmids and BACs were obtained from BacPac resources, DNA co-ordinates are hg38

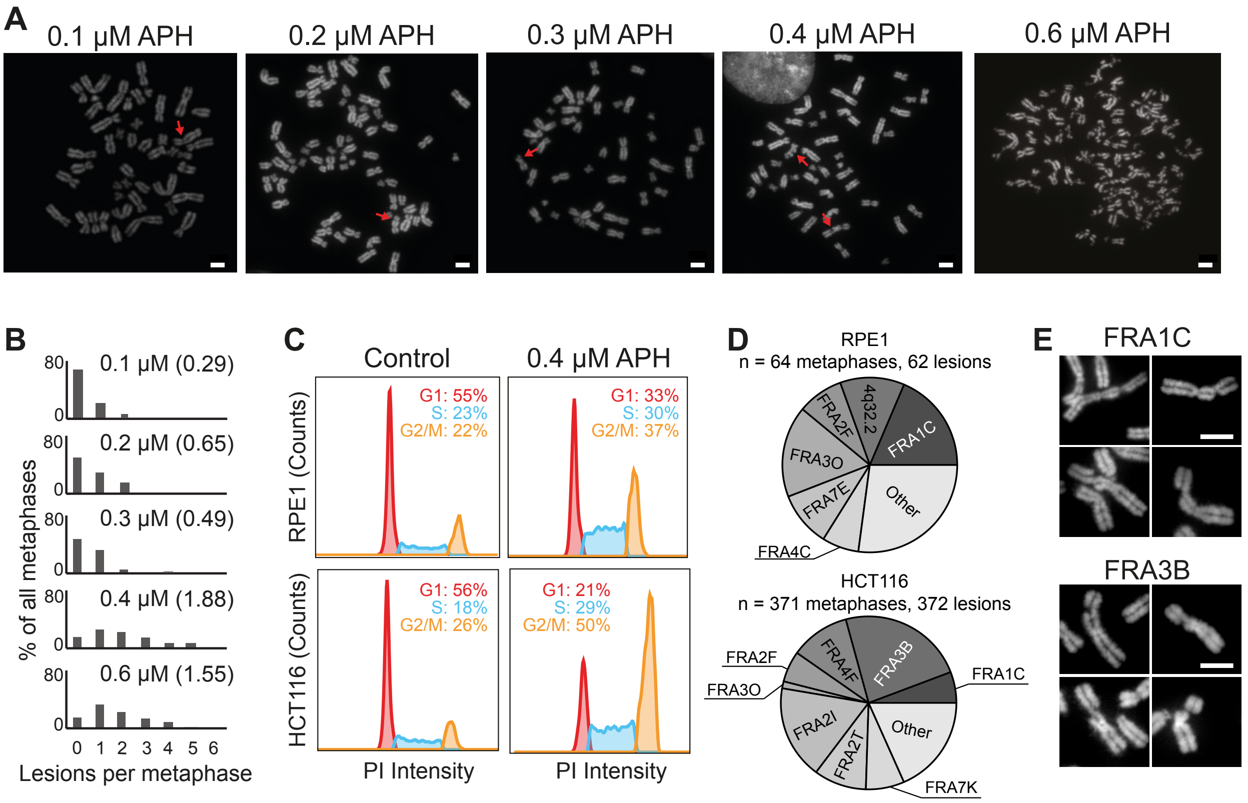

Supplementary Figure 1: Aphidicolin induced fragility in HCT116 and RPE1 cell lines, Related to Figure 1

A. Representative metaphases following treatment of HCT116 cells with different concentrations of aphidicolin (APH) for 24 hours. Lesions are indicated by red arrows. Right, metaphase showing multiple regions of incomplete axial compaction following treatment with 0.6 μM APH. Scale bar, 5 μm.

B. Proportions of HCT116 metaphase spreads showing different numbers of lesions following treatment with 0.1, 0.2, 0.3, 0.4 or 0.6 μM APH for 24 hours. Average breaks per metaphase are given in brackets. A minimum of 52 metaphases were characterised per condition.

C. Cell cycle profiles in the HCT116 and RPE1 cell lines in control conditions and following treatment with 0.4 μM APH for 24 hours, analysed via flow cytometry. Proportions of cells in the different stages of the cell cycle.

D. Pie charts showing proportion of lesions occurring at different CFS locations after APH treatment. Number of metaphases analysed are shown.

E. Representative images of lesion morphologies at two different CFSs, FRA1C (top) and FRA3B (bottom). Scale bar, 5 μm.

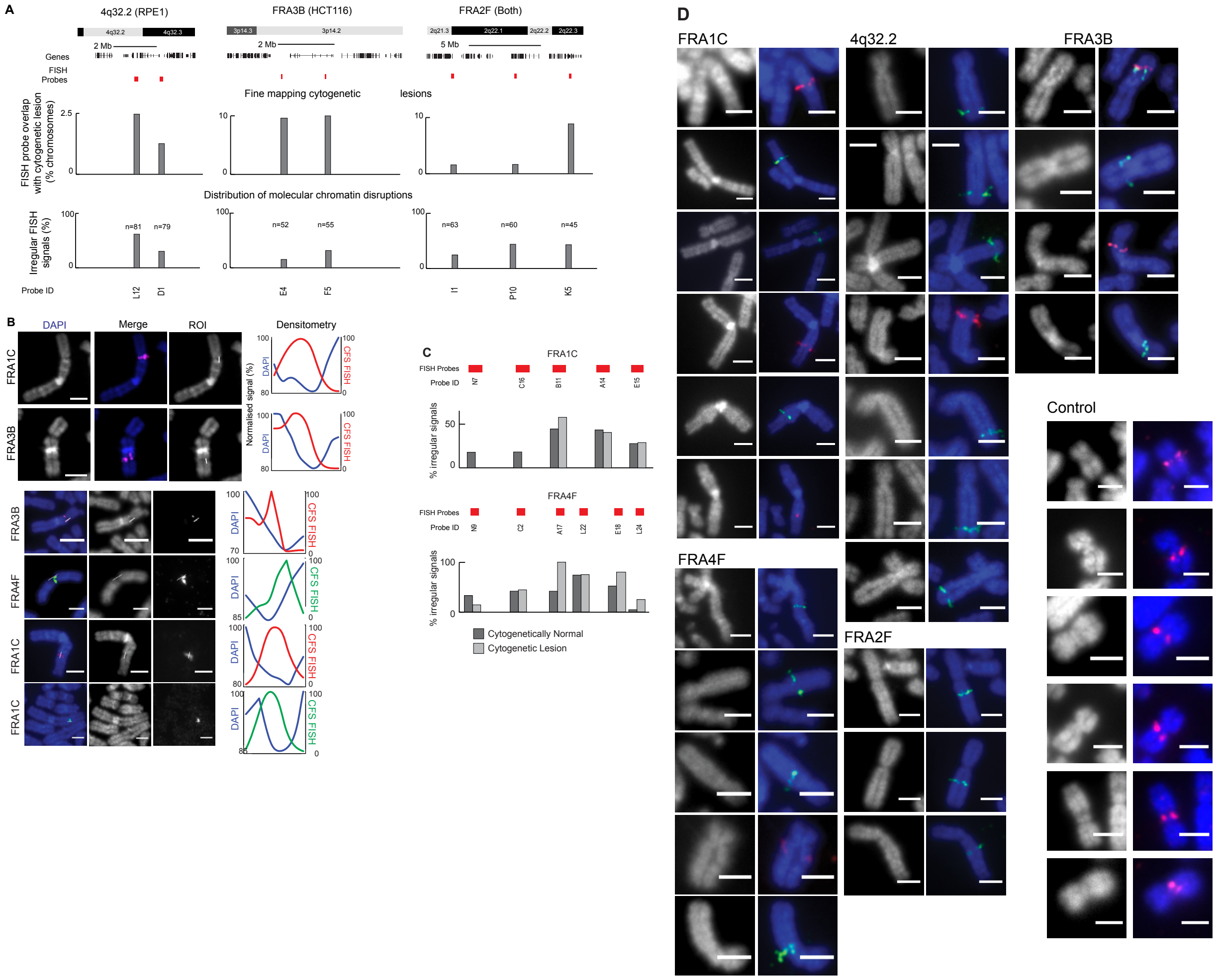

Supplementary Figure 2: CFS FISH probe signals across cytogenetic lesions and cytogenetically normal CFS regions, Related to Figure 2

A. Top, diagram showing FISH probes (red) spanning lesions at 4q32.2 (RPE1 cells), FRA3B (HCT116 cells) and FRA2F (Both cell lines). Bottom, probes were hybridised to metaphase spreads from cells treated with aphidicolin and counterstained with DAPI followed by quantification to fine map cytological lesions and distribution of molecular chromatin disruptions. Probe ID's (see methods) are shown.

B. Representative images showing FISH signals at CFSs FRA1C, FRA2F, 4q32.2, FRA4F and FRA3B. Quantification of FISH signal and DAPI signal marked by white line. Scale bar, 2.5 μm.

C. Quantification of irregular FISH signals at the FRA1C (RPE1) and FRA4F (HCT116) CFS sites in the absence (top) or presence (bottom) of cytogenetic lesions.

D. Images showing irregular FISH signals at common fragile sites (FRA1C, 4q32.2, FRA4F, FRA3B) but not at control loci. Scale bar, 2.5 μm.

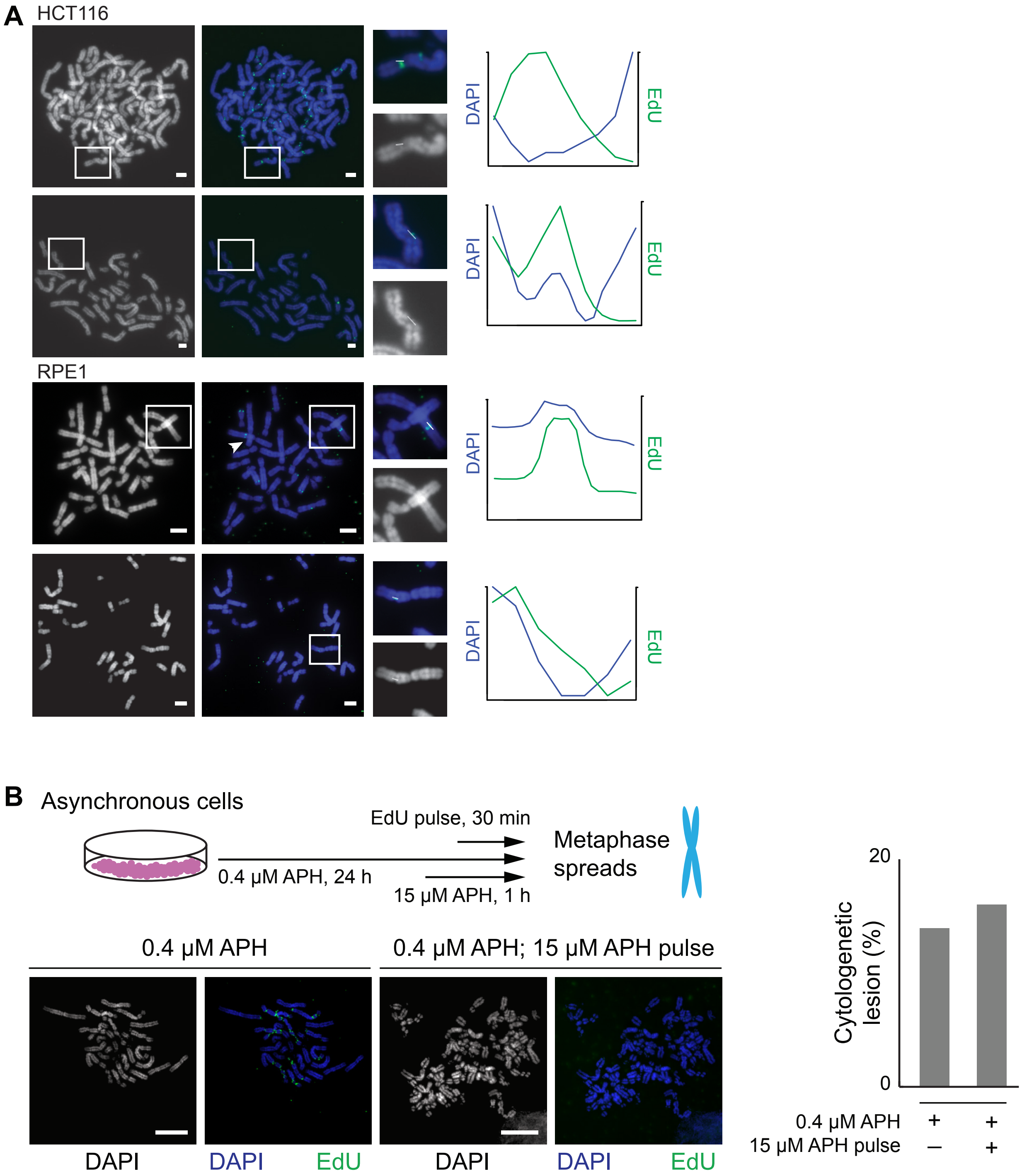

Supplementary Figure 3: MIDAS and chromatin decondensation following replication stress, Related to Figure 3

A. Representative images showing mitotic DNA synthesis in HCT116 and RPE1 cell lines following 24 h treatment with 0.4 μM APH. Inset, selected cytogenetic lesions with mitotic DNA synthesis. Right, intensity profiles of mitotic synthesis foci across the cytogenetic lesions. White lines indicate the regions selected for the intensity profile, produced in ImageJ. Scale bar, 2.5 μm.

B. Schematic of experiment to assess relationship between MIDAS and aberrant chromosome compaction. Cells were exposed to low concentration aphidicolin to induce replication stress and then high dose aphidicolin to inhibit DNA synthesis. Bottom left, representative metaphases showing MIDAS (EdU, green) in cells treated only with low dose aphidicolin and those treated with additional high dose aphidicolin to inhibit MIDAS. Right, quantification of metaphases showing chromosomal lesions in the presence or absence of a high dose APH pulse (n = 68 metaphases). Scale bar, 10μm.

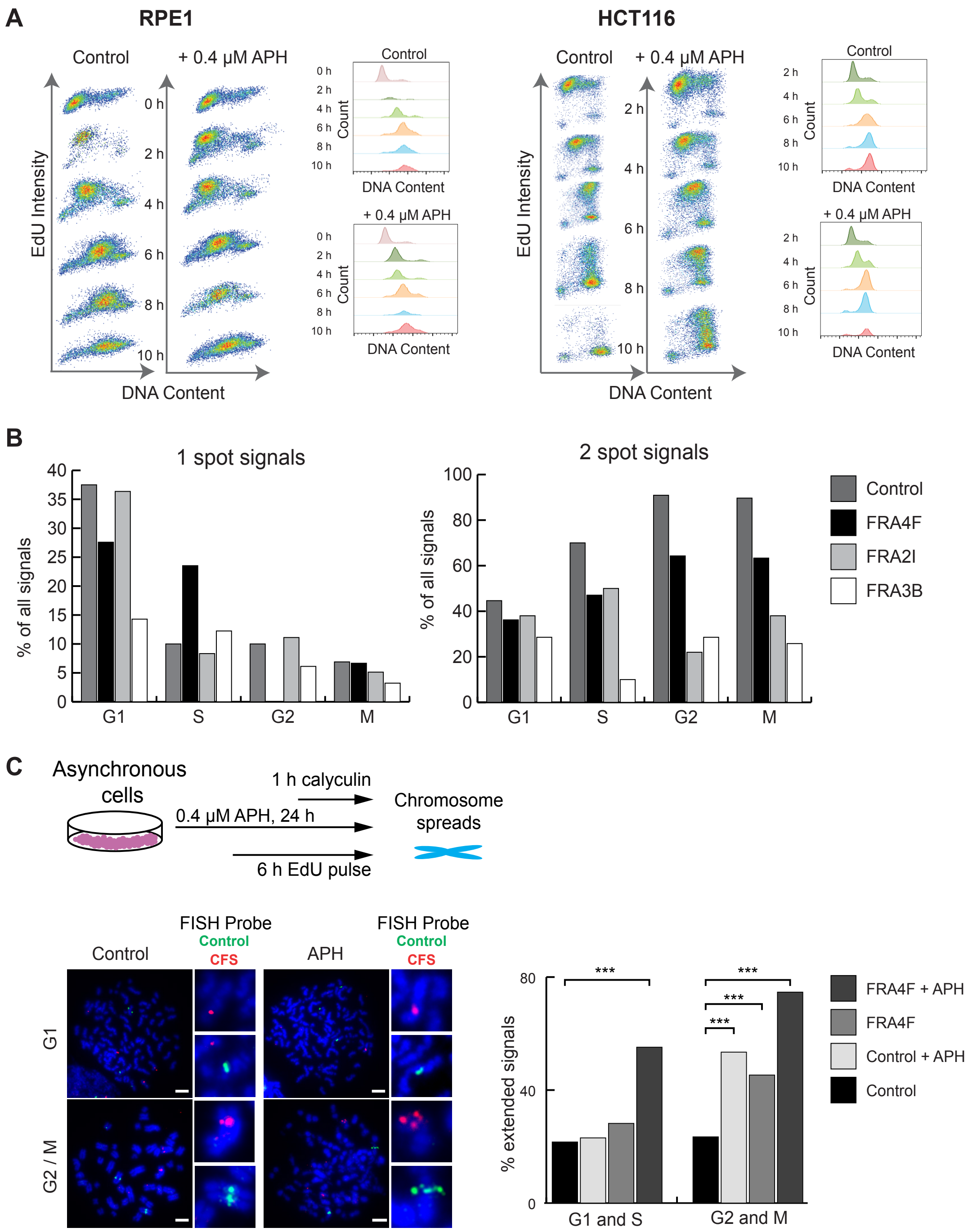

Supplementary Figure 4: Cell synchronisation and cell-cycle dependent signals following premature chromosome condensation, Related to Figure 4

A. Cell cycle analysis, using flow cytometry, at different time points after release from G1/S block, as described in Figure 4A. Samples were pulsed with EdU 30 min prior to harvesting to mark replicating cells. EdU intensity versus DNA content (left) and propidium iodide (PI) histograms of the cell populations (right) are shown for different time points.

B. Frequencies of one-spot and two-spot signals at CFS locations and a control, non- fragile location, across different cell cycle stages in prematurely compacted chromosomes, using calyculin.

C. Depiction of premature chromosome condensation (PCC) assay (see methods) in HCT116 cells. Cells treated with APH were labelled with EdU (6 h) and condensed using calyculin (1 h), harvested and hybridised to FISH probes for a control locus (11q13.2, probe P21) and CFSs (FRA4F, probe A17). Bottom left, representative chromosome images. Bottom right, quantification of irregular FISH probe signals at FRA4F (n = 207) and 11q13.2 control probe (n = 200); p-values for a $\chi 2$ test. Scale bars, 5 μm.

p values: ***$p < 0.001$.

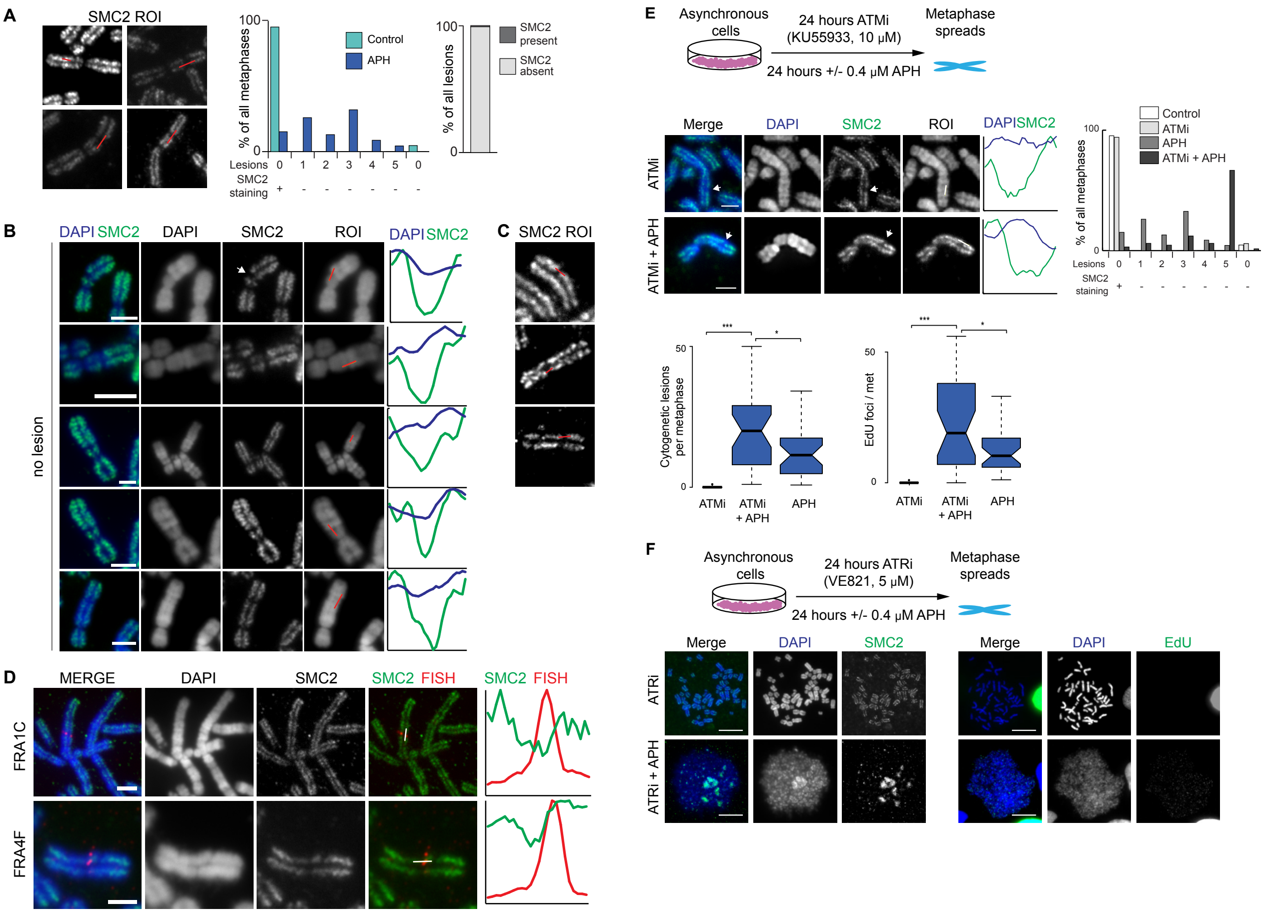

Supplementary Figure 5: SMC2 depletion at CFS loci on metaphase chromosomes, Related to Figure 5

A. Regions of interest encompassing regions depleted of SMC2 used for intensity measurements in Figure 5A. Right, frequency of cytogenetic lesions and lesion-free SMC2 depletion (last column) in the presence (n = 43 metaphases) or absence (n = 46 metaphases) of aphidicolin in HCT116 cells. Far right, quantification of SMC2 occupancy at cytogenetic lesions in HCT116 cells (n = 101 lesions).

B. Representative images and quantification of SMC2 depletion at cytogenetically normal chromosomes in RPE1 cells after aphidicolin treatment. Scale bar, 5 μm.

C. Regions of interest (red line) used for intensity measurements in Figure 5B.

D. Representative immuno-FISH image showing a FISH probe for the FRA1C or FRA4F locus overlap with regions of SMC2 depletion on metaphase chromosomes from RPE1 cells or HCT116 cells; right, intensity profiles across the region of interest indicated by white line. Scale bar, 2.5 μm.

E. Top, diagram depicting experimental procedure to analyse effect of ATM inhibition on chromosome structure. Middle left, representative images of cytogenetically normal chromosomes showing regions of SMC2 depletion following treatment with ATM inhibitor, and ATM inhibitor + aphidicolin. Scale bar, 2.5 μm. Middle right, frequency of cytogenetic lesions and lesion-free SMC2 depletion (last column) following ATM inhibition (n > 30 metaphases / condition). Bottom, quantification of the number of cytological lesions (left) and MIDAS foci (right) in HCT116 cells following treatment with ATM inhibitor, aphidicolin and ATM inhibitor + aphidicolin (n > 30 metaphases / condition). P-values are for a Student's t-test.

F. Top, diagram depicting experimental procedure to analyse effect of ATR inhibition on chromosome structure. Bottom, representative images of chromosomes and fragmented metaphases in HCT116 cells following treatment with ATR inhibitor and ATR inhibitor plus aphidicolin, stained for SMC2 (left) or DNA replication (right). Scale bar, 5 μm.

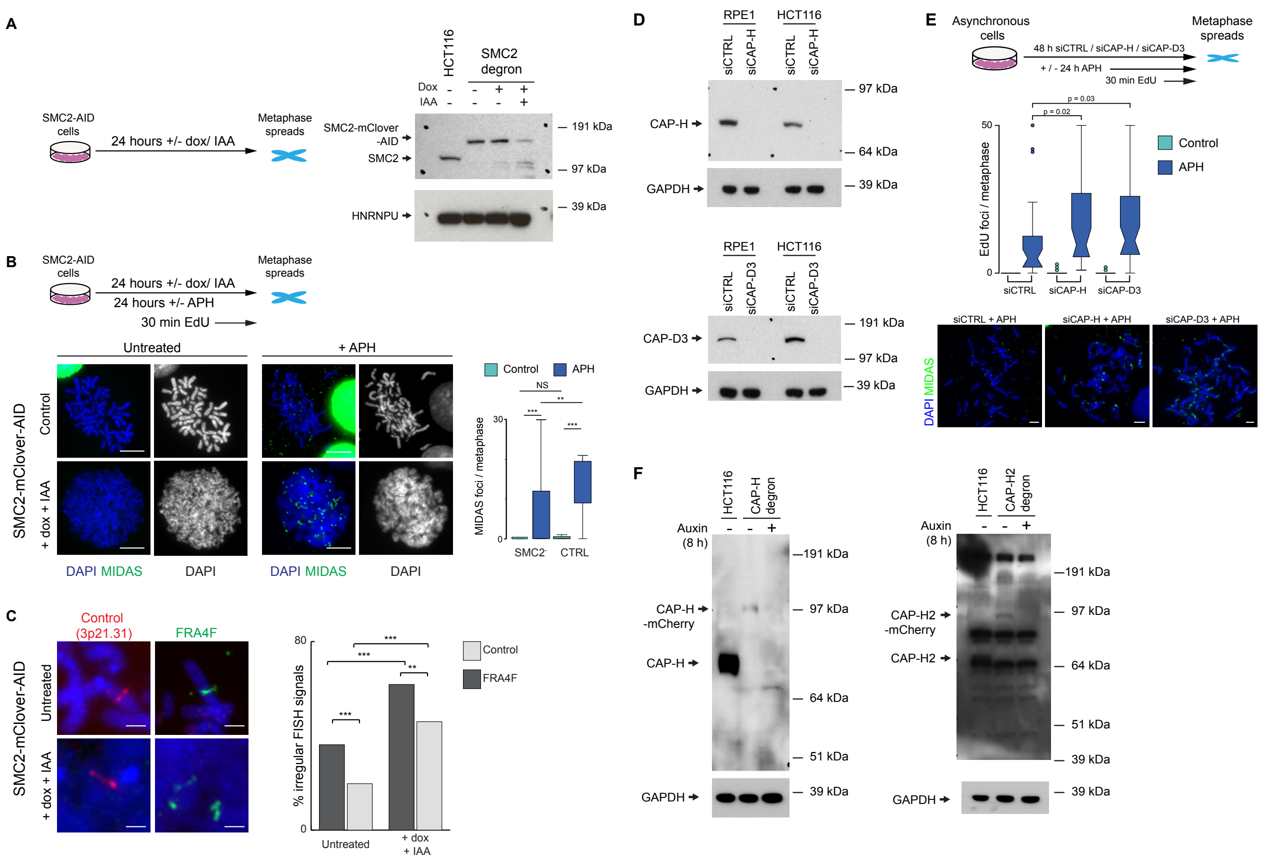

Supplementary Figure 6: Condensin depletion affects mitotic DNA synthesis and mitotic chromosome folding, Related to Figure 6

A. Left, diagram depicting auxin-induced SMC2 degradation in HCT116-SMC2-AID cell line. Right, western blot showing SMC2 degradation after auxin treatment.

B. Top, diagram depicting auxin-induced SMC2 degradation in HCT116-SMC2-AID cell line in the presence and absence of APH and EdU. Bottom left, Metaphase chromosome morphology and MIDAS labelling in the presence and absence of aphidicolin before and after auxin-induced SMC2 degradation. Scale bars, 5 μm. Bottom right, quantification of MIDAS foci per metaphase after SMC2 degradation (auxin) and APH treatment. P-values are for a Student's t-test.

C. Left, representative images showing FISH signals at the FRA4F fragile site and a control, non-fragile location in the HCT116-SMC2-AID cell line before and after auxin-induced SMC2 degradation. Scale bar, 2.5 μm. Right, quantification of the frequency of abnormal FISH signals before and after SMC2 degradation (n > 50 chromosomes / condition). P-values are for a χ^2 test.

D. Western blot showing depletion of CAP-H and CAP-D3 following 48 hours of siRNA treatment.

E. Top, diagram depicting RNAi-induced condensin degradation in HCT116 cells in the presence and absence of APH and EdU. Middle, boxplot showing the frequency of MIDAS foci per metaphase in HCT116 cells following CAP-H and CAP-D3 depletion in the absence (green) or presence (blue) of aphidicolin (n > 20 metaphases / condition). P-values are for Student's t-test. Bottom, representative images showing MIDAS in control and condensin depleted cells. Scale bars, 5 μm.

F. Western blot showing depletion of CAP-H and CAP-H2 following 8 hours of auxin treatment in HCT116 and HCT116 degron cell lines.
